# A New Challenge to Species Delimitation: Remarkable Genomic and Ecological Diversity in the Butterfly *Melitaea diamina*

**DOI:** 10.1111/mec.70474

**Published:** 2026-07-22

**Authors:** Loukia Spilani, Valéria Marques, Cecilia Montiel‐Pantoja, Miguel Sanjurjo‐Franch, Isabel Martínez‐Pérez, Sergio Montagud Alario, Leonardo Dapporto, Vlad Dincă, Roger Vila

**Affiliations:** ^1^ Institut de Biologia Evolutiva (CSIC‐Universitat Pompeu Fabra) Barcelona Spain; ^2^ Faculty of Biology University of Barcelona Barcelona Spain; ^3^ Independent Researcher Asturias Spain; ^4^ Independent Researcher Oviedo Asturias Spain; ^5^ Museu de la Universitat de València d'Història Natural (MUVHN) Universitat de València Burjassot Valencia Spain; ^6^ Dipartimento di Biologia dell'Università degli Studi di Firenze Florence Italy; ^7^ Ecology and Genetics Research Unit University of Oulu Oulu Finland; ^8^ “Grigore Antipa” National Museum of Natural History Bucharest Romania

**Keywords:** gene flow, host plant, phylogeny, population genomics, subspecies

## Abstract

Evolution relentlessly challenges any concept of species, and human efforts to fit a taxonomic hypothesis to patterns in nature often seem unsatisfactory. Genomics show that a solution does not always come with more data: a fine‐scaled view may reveal populations with a more gradual array of differentiation, infer cases of hybrid origin and bring to light the existence of ghost populations. The false heath fritillary, *Melitaea diamina*, is a Palearctic butterfly with patchy, localized populations highly dependent on humid temperate habitats. Despite its extensive geographic range, populations at the southern edge of its European distribution are scarce and poorly studied genetically. Here, we employed genome‐wide data to reveal the existence of two major groups in *M. diamina*: an Iberian and a Eurasian lineage. Within Iberia, we documented a remarkable diversity: the populations in the Pyrenees are of hybrid origin between the Eurasian lineage and a now apparently extinct Iberian population. The ghost population was related to three extant additional Iberian lineages that align with mitochondrial differentiation, distinct morphological traits, and/or ecological specialization in the form of host plant association. Gene flow analyses suggested historical admixture despite current isolation, underscoring the complex evolutionary history of these populations. We attempt a revised taxonomic framework for this novel array of evolutionary significant units, adopting an updated subspecies concept—defining subspecies as incompletely separated lineages within a more inclusive lineage. Such an approach allows accurate representation of evolutionary complexity without prematurely elevating these populations to species rank, thereby informing targeted, biologically meaningful conservation strategies.

## 1 Introduction

Although extensive debate has surrounded the concept of species—its precise definition and the criteria determining when two lineages have diverged sufficiently to merit recognition as separate species—comparatively less attention has been paid to a growing phenomenon observed with the advent of genomic data. Genome‐wide analyses frequently uncover lineages that are consistently differentiated genetically, and often ecologically or morphologically, yet still exhibit evidence of gene flow. In most cases, this gene flow occurs between sister species, but hybridization can also take place between substantially diverged species. Such cases are due to the persistence of incomplete pre‐mating barriers (Descimon and Mallet 2009). This complexity challenges traditional taxonomic frameworks, as such lineages are neither clearly conspecific nor fully reproductively isolated. While elevating these lineages directly to species rank might offer a straightforward solution, it often does not reflect the biological nuances involved, leading to inflated species counts and potential taxonomic instability.

Recent work has sought alternative ways to address these genetically distinct yet incompletely isolated lineages, proposing approaches that do not automatically elevate every newly identified lineage to species status (e.g., de Queiroz 2020; Dufresnes et al. 2023; Lukhtanov 2024; Vences et al. 2024). A recurring suggestion is to recognize some of these lineages as subspecies. Historically, subspecies were widely used to designate subtle morphological or ecological variants within geographically distinct populations. However, in the genomic era, the traditional subspecies concept has become somewhat neglected or replaced by genetically informed classifications, such as evolutionary significant units. de Queiroz (2020), among others, has recently proposed a modernized definition of subspecies, viewing them explicitly as “incompletely separated lineages nested within a more inclusive lineage.” Under this updated perspective, subspecies do not simply represent subordinate taxonomic ranks; rather, they reflect meaningful evolutionary units that are differentiated but still show evidence of ongoing or recent gene flow. Importantly, this approach demands rigorous evidence for lineage separation comparable to that required for species delimitation, while explicitly acknowledging incomplete isolation. This emerging conceptual framework may provide a more biologically meaningful and taxonomically stable way to recognize genomic lineages uncovered through modern population genomic analyses.

The false heath fritillary, *Melitaea diamina* (Lang, 1789), is a nymphalid butterfly widely distributed across the Palearctic. Despite its broad geographical range, it is patchily distributed in Europe, occurring mostly as localized populations, some of which may be abundant but geographically restricted. It is considered a weak flier (Wahlberg 1997), highly dependent on open habitats characterized by abundant larval host plants and scattered trees. Suitable habitats include humid meadows, pastures, woodland clearings, and marshy grasslands with sufficient sunlight and host plants. Habitat loss due to secondary vegetation succession, especially following the decline of grazing activities, represents a significant threat; in Fennoscandia, such habitat changes have already caused notable declines in local populations (Wahlberg 1997). Although several ecological studies have addressed aspects of its life history (Wahlberg 1997; Sanjurjo‐Franch et al. 2023), metapopulation dynamics (Hanski et al. 2000), and parasitoid prevalence (Kankare et al. 2005), genetic and genomic studies remain scarce.

Within Europe, larval host plants of *M. diamina* were traditionally considered exclusive to species of the genus *Valeriana*. However, recent work identified a novel larval host plant, *Centranthus lecoqii* Jord., a Mediterranean endemic, in a population from the Cantabrian Mountains of northern Spain (Sanjurjo‐Franch et al. 2023). Interestingly, this novel host plant association coincides geographically with the occurrence of a differentiated lineage previously identified by mitochondrial DNA (mtDNA) analyses (Dapporto et al. 2022). These analyses recovered two main mitochondrial lineages within Europe: a widespread lineage present across most of the species' range and a substantially diverged one (min p‐distance = 1.5%) restricted to two Iberian populations—one near Ports de Tortosa in Castelló and Tarragona, and another associated with *C. lecoqii* in the Cantabrian Mountains. In contrast, a neighbouring Cantabrian population does not share this differentiated mtDNA haplotype, nor does it share the newly reported host plant, but instead carries the common haplotype shared by populations throughout the rest of Europe (Dapporto et al. 2022; Sanjurjo‐Franch et al. 2023). A similar mitochondrial pattern was previously described by Dincă et al. (2015), although only the Ports de Tortosa population was identified at the time due to the smaller dataset analysed. While these mitochondrial datasets provide valuable preliminary insights, they are insufficient to comprehensively characterize the genetic diversity and fully resolve the population structure of *M. diamina*.

In this study, we leveraged genome‐wide single nucleotide polymorphisms (SNP) data generated through double‐digest restriction site‐associated DNA sequencing (ddRADseq; Peterson et al. 2012) to comprehensively investigate patterns of genetic diversity and population structure in *M. diamina*, focusing on the western part of its range. Motivated by earlier mitochondrial DNA and ecological studies that indicated the potential existence of cryptic differentiation within Iberia, our objectives were to: (1) characterize genomic variation across Iberian populations of *M. diamina* to clarify patterns of population differentiation and assess congruence or discordance with previously inferred ecological distinctions; (2) evaluate the extent of genetic admixture and potential reproductive isolation among these genomic lineages, thereby informing hypotheses about cryptic taxonomic diversity; and (3) propose a genomic‐based taxonomic framework prioritizing genome‐wide evidence to accurately represent evolutionary complexity and guide future conservation decisions.

## 2 Materials and Methods

### 2.1 Sampling

**FIGURE 1:**
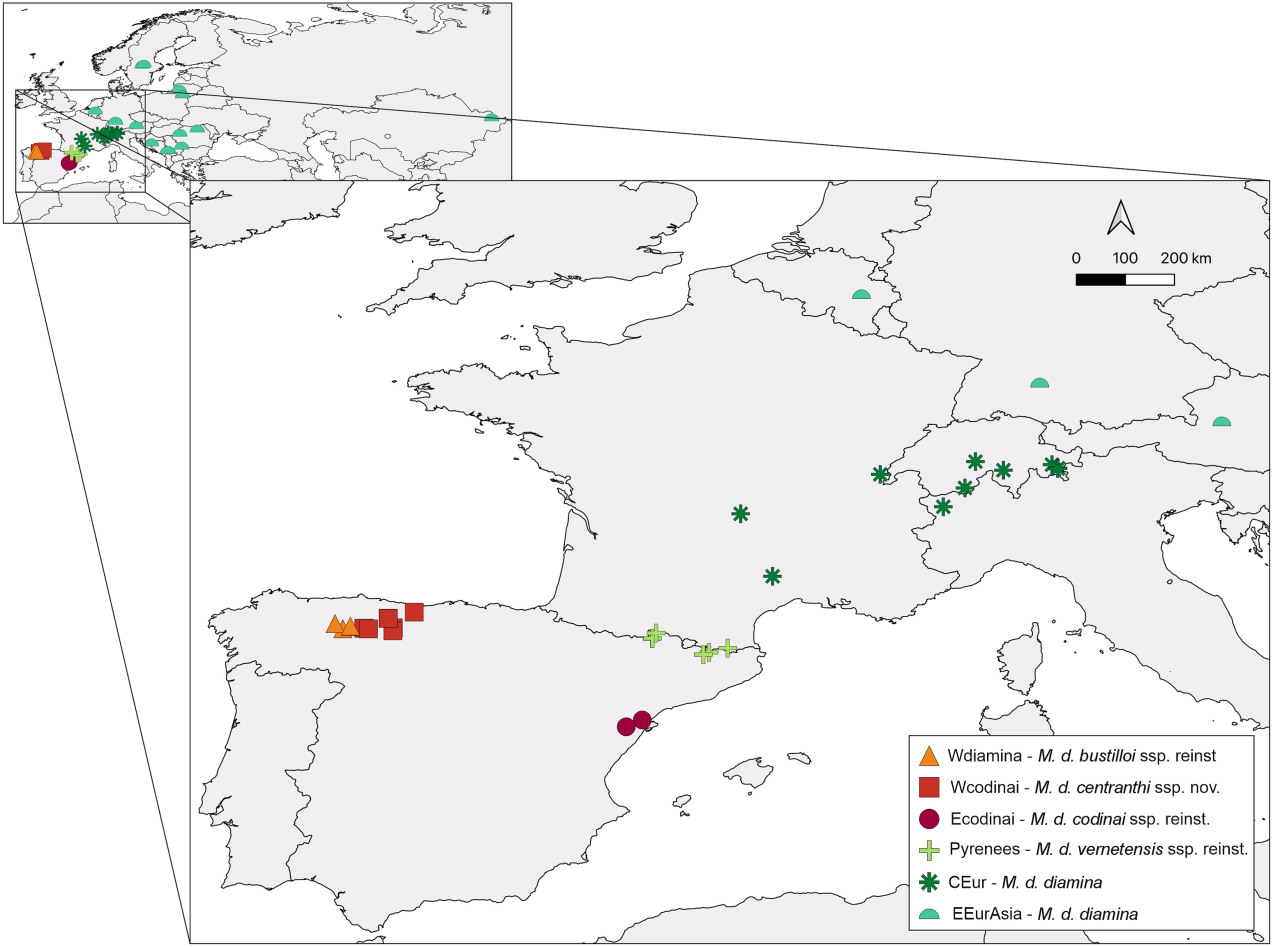
Geographical distribution of sampling sites. Colours indicate the different groups identified in the analyses.

*Melitaea diamina* has a wide distribution across the Palearctic, its range extending from Western Europe through Central and Eastern Europe, reaching Northern Europe. In Southern Europe, populations occur in northern Spain, France, Italy, and the Balkans, particularly in mountainous and subalpine regions. The species also extends eastward into Russia, the Caucasus, the Ural Mountains, western Siberia, and other parts of Asia, including Kazakhstan, Mongolia, and China. In this study, we aimed to cover most main populations in Europe, analysing a total of 66 samples. These included 64 specimens of *M. diamina* spanning the European part of its distribution, as well as 1 sample from Kazakhstan and 1 specimen of *M. protomedia*, used as an outgroup (Figure 1, Table S1). Butterfly bodies were preserved in 99% ethanol at −20°C, with wings stored separately as vouchers (Figures S7–S10).

### 2.2 ddRADseq Library Preparation

A ddRAD protocol was used to obtain genome‐wide representation. We extracted genomic DNA (gDNA) from half of the thorax using the DNeasy Blood & Tissue Kit (Qiagen). We checked the quantity of gDNA extracts using the PicoGreen kit (Molecular Probes). In order to increase gDNA quantity, we performed whole‐genome amplification using the REPLI‐g Mini Kit (Qiagen). We estimated again the concentration of the amplified gDNA with PicoGreen kit (Molecular Probes). For every sample, 500 ng of DNA were digested with 1 μL PstI, 2 μL MseI, and 5 μL of CutSmart Buffer (New England Biolabs), and water was added until a total volume of 50 μL. Samples were incubated for 2 h at 37°C and then frozen for enzyme deactivation. We did a purification step with AMPure XP magnetic beads (Agencourt) in a Biomek automated liquid handler (Beckman Coulter) with a final elution in 40 μL. DNA concentration was measured with PicoGreen, and this value was used for the pooling step. For ligation of the adapters used to later identify individuals, in every sample we added 5 μL T4 DNA Ligase Buffer (NewEngland Biolabs), 1 μL T4 DNA Ligase (New England Biolabs), 0.6 μL rATP (Promega), 5 μL P1 adapter (50 nM), 5 μL P2 adapter (50 nM), and 2.4 μL water. The P1 adapter included 45 unique Illumina primer sequences, 5 bp barcodes, and a TGCA overhang on the top strand to match the sticky end left by PstI. The P2 adapter included the Illumina primer sequences and AT overhangs on the top strand to match the sticky end left by MseI. It also incorporated a “divergent‐Y” to prevent amplification of fragments with MseI restriction sites on both ends. Ligation process was extended for 1 h at 22°C, and enzymes were deactivated at 65°C for 20 min. A total of 200 ng of each individual were pooled in tubes making three pools in three different tubes with a final volume of ~450 μL each. Every pool was purified with AMPure XP magnetic beads. Size selection was done at 300 bp with BluePippin (Sage Science). Finally, we performed PCR amplification, with primers RAD1.F (5′‐AATGATACGGCGACCACCGAGATCTACACTCTTTCCCTACACG ACG‐3′) and RAD2.R (5′‐CAAGCAGAAGACGGCATACGAGATCGT GATGTGACTGGAGTTCAGACGTGTGC‐3′). DNA was amplified in 60 μL volume reactions: 9 μL water, 30 μL Phusion High‐Fidelity PCR Master Mix (Finnzymes), 3 μL of each primer (10 mM), and 15 μL of DNA. Reaction conditions comprised a first denaturation at 98°C for 30 s, then 16 cycles of denaturation at 98°C for 10 s, annealing at 60°C for 30 s, and extension at 72°C for 40 s, with a final extension step at 72°C for 5 min. The PCR products were purified with AMPure XP magnetic beads, and DNA concentration was measured with PicoGreen. We measured size distribution and concentration of the pools with a Bioanalyzer (Agilent Technologies). Finally, we pooled libraries in equimolar amounts and sequenced them on an Illumina HiSeq X Ten platform using paired‐end 150 bp sequencing at Macrogen Inc. Europe (Amsterdam, the Netherlands). The demultiplexed fastq data are available under BioProject PRJNA1337706 stored in GenBank.

### 2.3 Data Cleanup and Filtering

Initial filtering steps, single‐nucleotide polymorphism (SNP) calling, and alignment were carried out using ipyrad v.0.9.102 (Eaton and Overcast 2020), using the publicly available genome of *M. diamina* (Wymann et al. 2025) as a reference. Sites with Phred scores below 33 were flagged as missing data. Reads with more than 5% missing sites, consensus sequences supported by fewer than 10 reads, or sites where an individual appeared to have more than two alleles were discarded for that individual. Filtering also excluded loci exceeding a maximum read depth threshold and those exhibiting excessive heterozygosity (> 5%) to mitigate potential paralogous loci. The minimum number of samples per locus was set to four, and the minimum trimmed length was 70 bp. The dataset obtained by ipyrad had a total of 27,880 SNPs before further filtering. For subsequent analyses, we selected loci and SNP datasets according to their specific requirements. Further filtering was applied to the SNP dataset using a custom R pipeline, integrating functions from SNPfiltR (DeRaad 2022) and vcfR (Knaus and Grünwald 2017) to iteratively remove low‐quality samples and genotypes following O'Leary et al. (2018). Missing data thresholds ranged from 98% to 70%, decreasing in 2% increments across iterations. Non‐biallelic SNPs were removed, and a minor allele count (MAC) filter excluded singletons and invariant sites arising post‐missingness filtering. To reduce SNP redundancy, linkage disequilibrium (LD) pruning was applied using the package SNPRelate (Zheng 2012), with an *r* ^2^ threshold of 0.2 within a 50 kb sliding window. Additionally, an alternative distance‐based thinning approach was available, ensuring SNPs were retained at a minimum spacing of 100 bp.

To generate datasets comparable across analyses requiring different data structures as input, we performed an additional round of locus assembly in ipyrad, retaining only individuals that passed the earlier SNP‐based filters. This second iteration produced whole‐locus sequence alignments (in phylip and loci formats) suitable for analyses that cannot use SNP matrices directly, such as the phylogenetic and network analyses described below, while ensuring reduced missing data and consistency in the individuals included across all analyses. The minimum number of samples per locus was adjusted accordingly to reflect the missingness level achieved in the SNP dataset.

### 2.4 Population Structure Analyses

Patterns of population genetic structure were explored using both non‐model‐based analyses (Principal Component Analysis—PCA & Discriminant Analysis of Principal Components—DAPC) and a model‐based hierarchical Bayesian clustering approach with STRUCTURE v.2.3.4 (Pritchard et al. 2000).

PCA and DAPC were performed using a custom R script. PCA was conducted with the ade4 package (Dray and Dufour 2007), after genotype data were mean‐imputed for missing values, centered, and scaled using the package adegenet (Jombart 2008). PCA retained the first three principal components (PCs), with individual scores visualized along axes 1–2 and 1–3. Groupings were assigned dynamically based on predefined location labels, and colours were mapped accordingly. DAPC was conducted using adegenet, following a clustering step that optimized the number of genetic clusters via the Bayesian Information Criterion (BIC). The optimal number of PCs retained in DAPC was determined through cross‐validation, with up to 500 PCs tested and 90% of the dataset used for training. Discriminant functions were retained dynamically, depending on the number of genetic groups identified. Results were visualized through custom scatter plots, and contingency tables were generated to compare group assignments across different clustering approaches.

For the STRUCTURE analysis, the number of clusters (K) varied from 1 to 8, with 20 replicate runs performed for each K. Each run consisted of a burn‐in period of 200,000 generations, followed by 1,000,000 MCMC iterations from which data were collected. STRUCTURE was run using a correlated allele frequency model with admixture (F‐model), which aims to identify clusters in approximate Hardy–Weinberg and linkage equilibrium. A hierarchical approach was implemented: after identifying the optimal K at the global level, the analysis was repeated separately on subsets of individuals grouped according to the initial results. Individuals with membership coefficients (Q‐values) less than a threshold of admixture (q < 0.9) were excluded from subsequent hierarchical analyses. To accelerate the analyses, Structure_threader (Pina‐Martins et al. 2017) was used, allowing multiple runs to be executed in parallel. The inference of K was evaluated by the ΔK method (Evanno et al. 2005) using STRUCTURE HARVESTER (Earl and von Holdt 2012) as implemented in Structure_threader. Additionally, CLUMPAK (Kopelman et al. 2015) was used to average results, define whether there is one or multiple run modes that generate consensus solutions allowing for label switching and testing for convergence and finally, to graphically compare the results across different K values via DISTRUCT (Jakobsson and Rosenberg 2007).

### 2.5 Phylogenetic Inference

To provide additional phylogenetic context for the population structure and gene flow analyses, we inferred a concatenated maximum likelihood (ML) phylogeny using IQtree v.2.3.6 (Minh et al. 2020) using the whole‐locus sequence alignment produced by the second ipyrad assembly described in Section 2.3. The full concatenated alignment was partitioned by locus using a nexus partition file, and the best‐fit substitution model for each partition was selected using ModelFinder (Kalyaanamoorthy et al. 2017) with the MFP + MERGE option, which additionally merges partitions with similar evolutionary dynamics to reduce model complexity. Branch support was assessed using 1000 ultrafast bootstrap replicates (Hoang et al. 2018). To complement the bootstrap support values and provide a measure of genealogical concordance that is less sensitive to large dataset sizes (Lanfear and Hahn 2024), we additionally calculated site concordance factors (sCF; Mo et al. 2023) on the resulting ML tree topology using 1000 random quartets per branch. The resulting tree, including both bootstrap support values and site concordance factors, is provided in Figure S5.

### 2.6 Gene Flow Analyses

To assess potential introgression events, we applied the ABBA–BABA test using Dsuite v.0.5.58 (Malinsky et al. 2021). This method evaluates allele frequency correlations across four populations to detect asymmetries indicative of gene flow. We used Dtrios to compute Patterson's D statistic (Patterson et al. 2012) and a block‐jackknife procedure to test whether D significantly differed from zero. Following Durand et al. (2011), we selected block sizes of 100 SNPs, ensuring they exceeded the extent of linkage disequilibrium in our dataset. The analysis was conducted using *M*. *protomedia* as a fixed outgroup, allowing us to identify asymmetric allele sharing between populations and test for significant introgression events. To further disentangle correlated signals of introgression identified by multiple four‐taxon tests, we applied the f‐branch metric introduced by Malinsky et al. (2018). This approach helps assign introgression events more specifically to particular branches of the phylogeny, aiding the interpretation of complex gene flow patterns across multiple populations.

Additional intraspecific gene flow events were investigated using the Julia package PhyloNetworks v.0.14.0 (Solis‐Lemus et al. 2017). This method infers reticulations by applying a maximum pseudolikelihood estimator to quartet concordance factors (CFs)—gene tree frequencies of four‐taxon trees—under the coalescent model, incorporating both incomplete lineage sorting and reticulation events (Solis‐Lemus et al. 2017). PhyloNetworks can also estimate the fraction of the genome potentially introgressed by retrieving inheritance values (γ), which represent the proportion of ancestral contribution to the hybrid lineage genome. To generate input trees, we used RAxML‐NG v.1.2.2 (Kozlov et al. 2019), applying the GTRGAMMA model with 500 bootstrap replicates per locus to estimate a tree for each locus, using the whole‐locus sequence alignments produced by the second ipyrad assembly described in Section 2.3. Concordance factors were then estimated in PhyloNetworks, with each individual assigned to a specific group corresponding to the genetic clusters identified by our previous PCA analyses. As a starting topology, we used a species tree inferred with ASTRAL v.5.7.8 (Zhang et al. 2018), built from the RAxML‐NG locus trees. We tested h‐values (number of gene flow events) ranging from 0 to 8, performing 50 independent SNaQ runs per h‐value to reduce the probability of retaining local optima. Optimal h‐values were evaluated using a slope heuristic based on the pattern of improvement in pseudolikelihood scores (negative log pseudolikelihood; net.loglik) as h increased (Solís‐Lemus and Ané 2016). Typically, a sharp improvement in pseudolikelihood is expected until h reaches the most suitable value, after which improvements become smaller and more linear. Although no definitive criterion exists to determine what constitutes a significant pseudolikelihood improvement, we leveraged this heuristic approach to identify the most biologically meaningful number of hybridizations.

For the ABBA‐BABA analysis, the Kazakh specimen was treated as a separate terminal from the remaining EEurAsia samples. This decision was motivated by the considerable sampling gap between Kazakhstan and the rest of the EEurAsia group and the tendency of the Kazakh specimen to differentiate from the remaining samples in the PCA and STRUCTURE analyses (see Results). Given that ABBA‐BABA tests are based on allele sharing, combining a specimen with a high degree of differentiation, further amplified by the absence of intermediate samples, with the EEur specimens risked obscuring the detection of gene flow events between that group and others. This separation was not applied in the PhyloNetworks analysis, which does not rely on allele sharing in the same direct way, and where the IQtree topology supports the inclusion of the Kazakh specimen within the broader EEurAsia group.

## 3 Results

### 3.1 Population Structure Analyses

**FIGURE 2:**
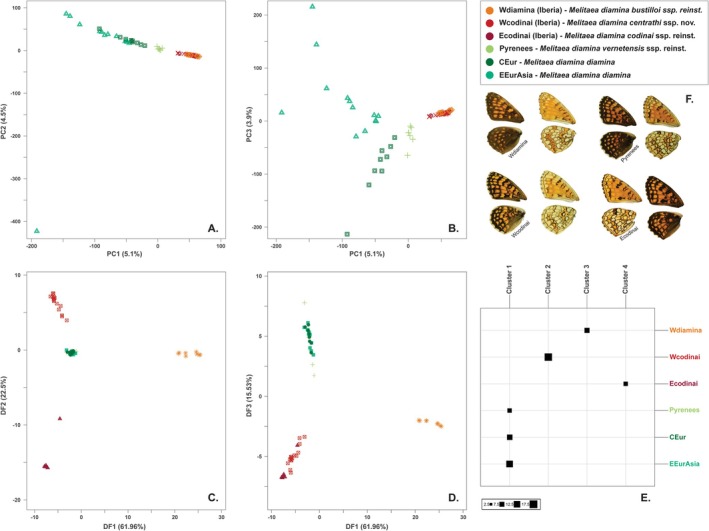
Population structure of *M*. *diamina* as inferred by Principal Components Analysis (PCA) and Discriminant Analysis of Principal Components (DAPC) on a dataset of 41,876 SNPs. (A) PCA plot of first two principal components (PCs); (B) PCA plot visualizing the 1st and 3rd PCs; (C) DAPC plot visualizing the first two discriminant functions (DFs); (D) DAPC plot visualizing the 1st and 3rd DFs; (E) Contingency table identified by k‐means clustering. Rows correspond to groups formed based on the groups identified by the rest of the analyses, while columns correspond to inferred groups. The size of the squares is proportional to the number of individuals; (F) Upperside and underside view of one individual from each of the focal subspecies. For a complete list of photos and their metadata see Table S1 and Figures S7–S10.

PC1 explained 5.1% of the observed variance, clearly separating the samples into two groups: those from the Iberian Peninsula and those from the rest of Europe and Kazakhstan, with the Pyrenean samples (Pyr) positioned between the two groups. PC2 accounted for 4.5% of the variance and exhibited a similar, albeit more subtle, separation pattern (Figure 2A), although the variance along this axis is largely driven by the single specimen from Kazakhstan. Given the large geographic gap between the Kazakh locality and the next easternmost samples within Europe (Figure 1), and given that the remaining samples appear arranged along a cline consistent with their geographic distribution, we consider this specimen's placement to be more reflective of undersampling in the region than of any distinct biological signal. PCA visualizations excluding this specimen, which more clearly reveal the underlying structure among the remaining samples, are provided in Figure S1. Visualization of PC1 and PC3 (Figure 2B) further distinguished non‐Iberian samples into Central Europe (CEur: France, Switzerland, Northern Italy) and Eastern Europe and Asia (EEurAsia: remaining European samples and Kazakhstan).

The optimal k‐means clustering solution for the DAPC (K = 4, Figure S2) grouped Pyrenean, Central European, Eastern European, and Kazakh populations together, while dividing Iberian samples into three distinct groups aligned with geography: Ports de Tortosa in Castelló and Tarragona (“Ecodinai”), western Cantabrian mountains (“Wcodinai”), and eastern Cantabrian mountains (“Wdiamina”) (Figure 2E). We refer to the Catalan population near Ports de Tortosa as “Ecodinai” and the Cantabrian population sharing the same mitochondrial haplogroup as “Wcodinai”, reflecting previous mitochondrial evidence indicating their close genetic relationship and the historical designation of the Ports de Tortosa locality as the type locality of *Melitaea diamina codinai*. As a result of the k‐means clustering result, three discriminant functions (DFs) were retained. When DF1 and DF2 were visualized together, a star‐like pattern emerged, with samples from Central and Eastern Europe and the Pyrenees clustered in the center, while the three Iberian populations were positioned around them, forming the points of the star, more or less equidistant from one another (Figure 2C). When DF1 and DF3 were visualized together, a triangle‐like pattern appeared, where the European‐Asian cluster formed one corner, the Ports de Tortosa population (Ecodinai) clustered with Wcodinai from the Cantabrian Mountains at the second corner, while Wdiamina, also from the Cantabrian Mountains, formed the third corner of the triangle (Figure 2D).

**FIGURE 3:**
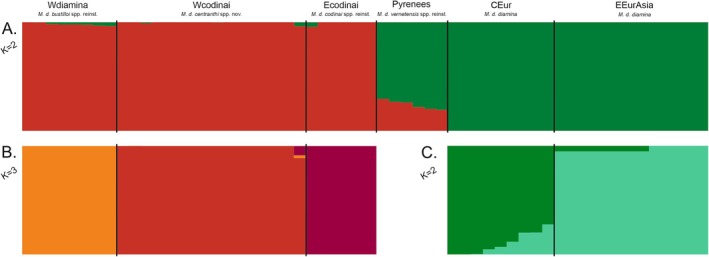
Population structure of *M*. *diamina* as inferred by STRUCTURE on a dataset of 41,876 SNPs. Black vertical lines indicate the different groups as identified by the set of analyses (A) Bar plot of individual *q*‐values for K = 2 for the entire dataset; (B) Bar plot of individual q‐values for K = 3 for the Iberian subset; (C) Bar plot of individual *q*‐values for K = 2 for the Eurasian subset.

The ΔK analysis performed on the STRUCTURE results supported the presence of two main clusters (K = 2; Figure S3), corresponding to the split between Central and Eastern Europe and Asia on one hand and Iberia on the other, with the specimens from the Pyrenees being admixed (Figure 3A). The hierarchical STRUCTURE analysis on non‐admixed individuals further revealed the separation of Central Europe from Eastern Europe and Asia (STRUCTURE on the European cluster; K = 2) (Figure 3C) and a clear differentiation of the Iberian populations into the three groups previously identified by k‐means clustering (Ecodinai, Wcodinai, and Wdiamina) (STRUCTURE on the Iberian cluster; K = 3) (Figure 3B). To further explore the robustness of the global clustering result, we examined the clustering solutions recovered across additional values of K (Figure S4). Although the ΔK plot (Figure S3) shows a clear primary peak at K = 2, consistent with the optimal solution identified above, secondary peaks at K = 3 and K = 5 are also present, a pattern typical of datasets with hierarchical population structure where the uppermost level of partitioning is captured first, followed by progressively finer subdivisions. The clustering solutions recovered at K = 3–6 are broadly congruent with the hierarchical results above, progressively resolving the same population groupings (Figure S4). At K = 3 and K = 4, the Pyrenean individuals are consistently recovered as admixed between the Iberian and Eurasian lineages in the most frequently occurring clustering solutions. From K = 5 onwards, the Pyrenean individuals tend to be assigned their own distinct cluster. Across all values of K, Wcodinai and Ecodinai are consistently recovered together in the most frequently occurring clustering solutions, with Wdiamina resolved as a separate group from K = 4 onwards. While alternative clustering solutions do exist at higher values of K, reflecting different combinations of the three Iberian populations across configurations, these are less frequently recovered and do not alter the overall picture of a close relationship between Wcodinai and Ecodinai.

### 3.2 Phylogenetic Inference

The concatenated maximum likelihood phylogeny inferred by IQtree, together with the ASTRAL species tree produced as the starting topology for the PhyloNetworks analysis (Section 2.6), are provided in Figures S5 and S6. Both trees recover two major clades: one comprising the Iberian populations and the Pyrenees, and the other comprising the Central and Eastern European and Asian samples. Within the Iberian clade, both trees recover Wcodinai and Ecodinai as sisters, with Wdiamina branching subsequently. The two topologies differ in two respects that reflect methodological constraints rather than genuine incongruence. First, in the IQtree topology the Pyrenean samples are recovered as paraphyletic with respect to the rest of the Iberian clade, whereas in the ASTRAL tree they form a monophyletic group, as individuals were required to be assigned to predefined groups prior to analysis. Second, the Kazakh specimen is recovered as basal to the Eastern European clade with a notably longer branch in the IQtree topology, a level of resolution that is similarly not visible in the ASTRAL species tree for the same reason.

### 3.3 Gene Flow Analyses

**FIGURE 4:**
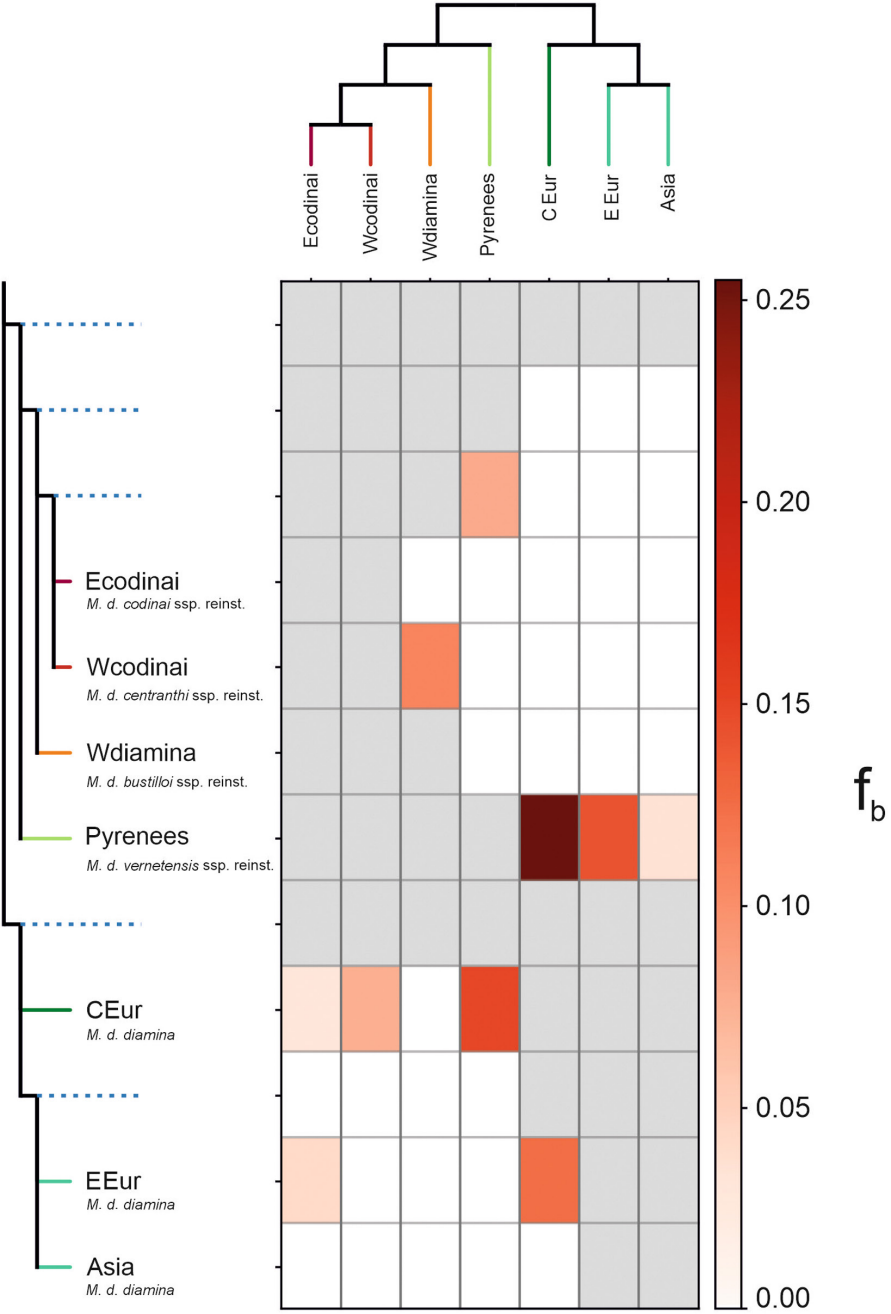
F‐branch analysis on a dataset of 44,S414 SNPs indicating ancestral introgression between the Pyrenees and the ancestor to the rest of the Iberian groups (Wdiamina, Wcodinai & Ecodinai), Pyrenees and Eurasia, Wdiamina and Wcodinai and eastern Europe with central Europe and Ecodinai. *Melitaea protomedia* was used as a fixed outgroup.

The ABBA–BABA test revealed significant gene flow among various groups (Figure 4). As observed in previous analyses, individuals from the Pyrenees population represent an admixture between the Iberian and European lineages. Interestingly, the Iberian genetic contribution to the Pyrenean population likely originates from an ancestral lineage common to the entire Iberian clade, suggesting that this introgression event occurred deeper in the past. Additionally, a low but detectable level of admixture between the central European group (CEur) and populations within the Iberian Peninsula (Ecodinai, Wcodinai, Pyrenees) was identified. Finally, evidence of admixture was also observed between the neighbouring Wcodinai and Wdiamina populations in northern Iberia.

**FIGURE 5:**
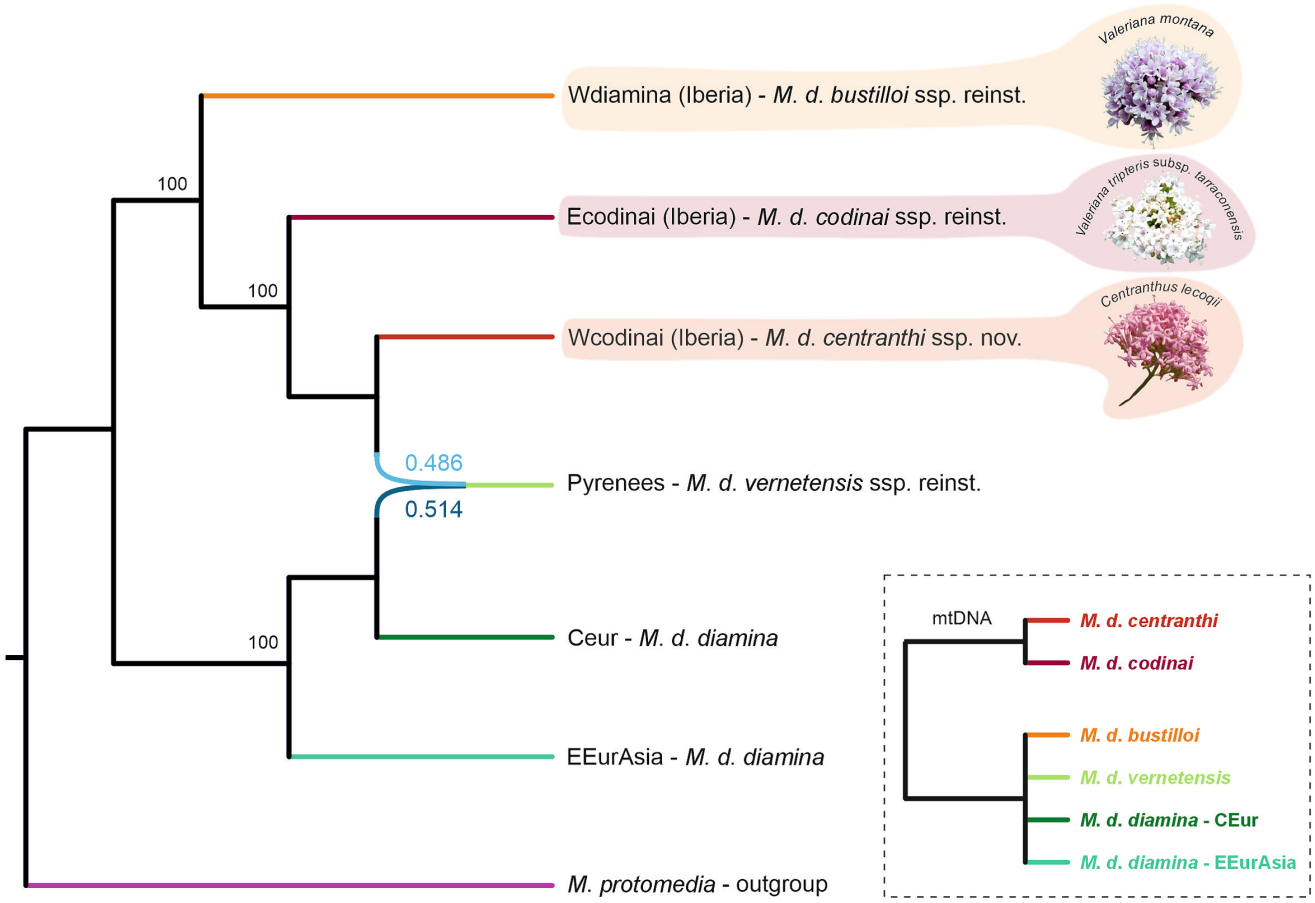
Phylogenetic network estimated with the Species Networks applying Quartets (SNaQ) pipeline with one hybridization event. The blue edges denote the hybridization event identified, with numbers next to the edges denoting the proportion of genes that were transferred from each lineage. The inset shows a simplified representation of mitochondrial DNA relationships among the same groups, adapted from Dapporto et al. (2022).

PhyloNetworks inferred a single introgression event between the Pyrenees and an unsampled population closely related to Wcodinai (Figure 5). Methodological constraints, such as PhyloNetworks' inability to detect gene flow between sister lineages and restrictions to independent, non‐overlapping reticulations, may have limited detection of additional introgression events.

## 4 Discussion

### 4.1 Understanding the Population Structure of *M. diamina*

This study indicates the presence of two major groups within *Melitaea diamina*: a Eurasian group and an Iberian group, with their contact zone situated in the Pyrenees. Considering its limited dispersal ability and its preference for cool, submontane habitats, it is likely that, particularly during the current interglacial, the main barriers to gene flow of this species are represented by the longitudinal valleys of the Iberian Peninsula rather than by the mountain ranges (Dapporto et al. 2024). An opposite pattern may have occurred during the Quaternary glacial maxima, when the Pyrenees likely became a barrier that was difficult to cross. The complex dispersal patterns during the Quaternary, together with the arrangement of the main Iberian mountain chains—separated by major valleys, unlike the continuous Alpine–Apennine and Dinaric–Balkan systems—may, in this case as well, underlie the well‐known phenomenon of “refugia within refugia” (Schmitt 2007).

Within each major group, further subdivisions were identified by our hierarchical approach. Specifically, the Eurasian cluster separated into two genetically distinct subgroups (CEur and EEurAsia), displaying an admixture cline consistent with geographic expectations, particularly evident in the area including Switzerland, Belgium, Germany, and Austria. The taxon diamina was described from Augsburg, Bavaria, Germany and, according to our results, this population is close to the area of admixture between the CEurope and EEurope‐Asia lineages. Conservatively, we treat both of these lineages as the nominotypical subspecies M. diamina diamina, although this hypothesis may be revised in the future. Indeed, if sampling were extended further eastward, it is likely that additional geographically structured clusters and corresponding admixture zones would emerge, potentially forming a more linear, stepwise pattern of differentiation extending across the species' range to its easternmost populations in Korea.

The admixed nature of the Pyrenean population, clearly captured at lower values of K in the STRUCTURE analysis, is further reflected in the paraphyletic placement of Pyrenean samples in the IQtree concatenated ML phylogeny, where different individuals appear to carry different proportions of Iberian and Eurasian ancestry. The progressive disappearance of this admixture signal at higher values of K is most likely a methodological artifact rather than a biological one, driven by the simultaneous fragmentation of the parental clusters into finer‐scale units and the accumulation of post‐admixture differentiation within the Pyrenean population itself, both of which make it increasingly difficult for the algorithm to represent mixed ancestry through shared coefficients across two parental clusters. Taken together, the STRUCTURE, IQtree, PhyloNetworks, and ABBA‐BABA analyses converge on a consistent picture of the Pyrenean population as a hybrid lineage situated at the secondary contact zone between the Iberian and Eurasian groups. Both PhyloNetworks and ABBA‐BABA analyses indicate that the admixed ancestry of the Pyrenean population traces back to a lineage that is no longer represented in our sampling, whether through extinction or incomplete geographic coverage. While the two methods differ in the implied timing of this event, this discrepancy is expected given that they operate under fundamentally different statistical frameworks and that the introgression signal in the data likely reflects a geographically extended rather than instantaneous process. The more robust takeaway is therefore the shared inference of a ghost lineage, which is consistent across both approaches regardless of the precise timing.

Within the Iberian Peninsula, three distinct populations were identified, corresponding broadly to the three main sampling regions: Ports de Tortosa in Castelló and Tarragona (Ecodinai), and two neighbouring populations in the Cantabrian Mountains (Wcodinai & Wdiamina). Interestingly, despite their geographic separation, the two populations sharing a mitochondrial haplotype (Ecodinai and Wcodinai) are consistently recovered as sisters across multiple independent lines of evidence—the ASTRAL species tree, the IQtree concatenated ML phylogeny, the DAPC DF1–DF3 visualization, and the major clustering solutions of the global STRUCTURE analysis (Figures 2, 3, 5; Figures S5 and S6)—with Wdiamina branching subsequently in each case. This is particularly noteworthy given that Wcodinai and Wdiamina are geographically much closer to one another, pointing to a shared evolutionary history between Ecodinai and Wcodinai that transcends their current geographic isolation.

### 4.2 Insights Into the Iberian *M. diamina* Populations

The Iberian Peninsula appears particularly suitable for harboring differentiated genetic lineages, due in part to its relative geographic isolation, which is further reinforced by the Pyrenees acting as a barrier to gene flow (Dapporto et al. 2024). In the case of *Melitaea diamina*, a butterfly primarily distributed across temperate regions of Europe, this geographic configuration could be significantly limiting the genetic exchange between Iberian populations and those located further east. Within the Iberian Peninsula itself, despite the comparatively small geographic scale, two populations of *M. diamina* show clear ecological and morphological differentiation in the Cantabrian Mountains. Sanjurjo‐Franch et al. (2023) noted that, despite extensive fieldwork between 2018 and 2023, these two populations were never observed in each other's habitat, even though they are relatively close geographically. Additionally, these populations have evolved distinct ecological and morphological traits, most notably in their host plant usage. One population (Wcodinai) relies exclusively on the recently identified host plant *Centranthus lecoqii*, a Mediterranean endemic. The neighbouring population (Wdiamina) uses *Valeriana montana* at unusually high elevations with rocky environments. Interestingly, despite their current ecological and morphological differentiation, our gene flow analyses suggest these two populations were probably in contact at some point in their past.

The population near Ports de Tortosa in Castelló and Tarragona exhibits distinct ecological specialization, exclusively utilizing a narrowly distributed subspecies, *Valeriana tripteris* ssp. *tarraconensis*. Interestingly, although *Centranthus lecoqii* is also present near Ports de Tortosa and in neighbouring regions, extensive field surveys indicate that the butterfly does not utilize this plant locally. Despite thorough fieldwork, no larvae associated with *Centranthus* were identified in or around Ports de Tortosa. The authors also searched suitable habitats for the butterfly in adjacent regions, particularly areas where Valeriana tripteris ssp. tarraconensis has been recorded, such as Castellón and Teruel. Although habitat conditions appeared suitable, these surveys did not detect any new population of *M. diamina*. Finding additional southern or western populations, geographically distant from currently known localities, remains a compelling possibility—particularly given results obtained through the gene flow analyses, which suggest historical connections or gene flow events involving currently unsampled or extinct populations.

Historical records combined with recent first‐hand observations by one of the authors indicate a severe decline in the Ports de Tortosa population of *M. diamina*. After initially discovering a male butterfly at this locality, it took one of the co‐authors 12 years to locate additional individuals, highlighting the population's extreme scarcity. Subsequently, following the sampling of specimens included in this study, extensive field surveys conducted by the same author—covering multiple expeditions, numerous forest tracks, and suitable habitats—have yielded only a single additional individual. Moreover, visits to other historically documented localities cited by Sagarra i de Castellarnau (1930) and De‐Gregorio (2001) have failed to detect the species. Even at Monte Caro, a site where adults and larvae had previously been recorded, recent visits have not produced any new observations. These findings strongly indicate that this once stable and well‐established population is now critically endangered. Given that the Ports de Tortosa locality is remote and largely free from direct human disturbances, its alarming decline is likely driven by climatic or other subtle environmental changes. Positioned at the ecological margin and representing one of the southernmost limits of the European distribution of *M. diamina*, this population—and indeed all Iberian populations—should be considered highly vulnerable to habitat loss and climate change. Peripheral populations with distinct genetic and ecological features—often inhabiting climatically marginal or topographically isolated environments—are increasingly being recognized as important conservation targets, even when overlooked by traditional taxonomic or conservation frameworks. Comparable examples include *Erebia epiphron* (Knoch) (Hinojosa et al. 2019), *Erebia pandrose* (Borkhausen) (Sistri et al. 2022), and *Lasiommata petropolitana* (Fabricius) (Bonifacino et al. 2022), among others. Although *M. diamina* is currently listed as Least Concern both in Europe and the Mediterranean region, our findings demonstrate how genomics, combined with ecological and field‐based data, can uncover previously unrecognized population declines, offering a more robust basis for reassessing conservation priorities. In this context, it becomes especially urgent to translate such reassessments into targeted conservation actions—particularly those aimed at maximizing habitat heterogeneity, which is emerging as a key resource for enabling butterflies to persist under accelerating climatic change (Bruschini et al. 2024; Hayes et al. 2024).

### 4.3 Taxonomic Evaluation and Reconsiderations

Given the genetic differentiation uncovered in this study, as well as the evidence we have for ecological and morphological differentiation among the Iberian populations, it is crucial to evaluate their conservation status carefully and ensure that taxonomic decisions adequately reflect this complexity. Although recognizing these Iberian lineages as separate entities based on genomic differentiation might seem straightforward, such an approach could oversimplify their complex biological relationships. Notably, the Pyrenean population is clearly admixed between Iberian and European lineages, indicating incomplete reproductive isolation, at least in some past colder period when the clades were likely more widely distributed and possibly parapatric. Within the Cantabrian Mountains, the geographically close Wcodinai and Wdiamina populations show genomic and mitochondrial differentiation despite evidence of historical gene flow. Furthermore, these populations differ markedly in habitat preferences, morphology, and larval host plant associations, with Wcodinai specifically utilizing *Centranthus lecoqii*, whereas Wdiamina uses *Valeriana montana*. Interestingly, although Wcodinai shares its mitochondrial haplogroup with the geographically distant Ecodinai population near Ports de Tortosa—which is present in similar habitats (calcareous ravines and scree slopes)—these two lineages differ in the host plant, as the latter exclusively uses a narrowly distributed *Valeriana tripteris* ssp. *tarraconensis*. This discordance between genomic relatedness and host plant specialization underscores the complex evolutionary history of these Iberian populations. Therefore, while genomic evidence could support the elevation of Ecodinai + Wcodinai to species level, the case of the puzzling lineage of Wdiamina makes this an inadequate solution.

Given the current state of knowledge about this group of taxa and following the updated definition of subspecies recently proposed by de Queiroz (2020), we argue that this particular case fits his modernized subspecies concept, where subspecies represent incompletely separated lineages nested within a more inclusive lineage. In this context, the naming conventions for these incompletely separated lineages become representational devices, rather than rigid taxonomic ranks, as discussed by de Queiroz (2020). According to his modernized concept, the use of trinomials clearly conveys incomplete lineage separation, whereas binomials would require additional annotation to indicate this incompleteness explicitly. Either approach can effectively represent the nuanced biological reality of these populations. Considering the scientific names available, the population from Ports de Tortosa in Castelló and Tarragona retains the subspecific name *Melitaea diamina codinai* ssp. reinst. For the Cantabrian populations, only one previously established subspecies name exists. Therefore, we propose naming the population previously referred to as Wcodinai as *Melitaea diamina centranthi* ssp. nov. highlighting its specialized host plant association, while reinstating the name *Melitaea diamina bustilloi* ssp. reinst. to the Wdiamina population. Finally, we propose treating the admixed populations from the Pyrenees as ssp. *vernetensis* in agreement with some previous publications (Tolman and Lewington 1997; Tshikolovets 2011; García‐Barros et al. 2013). This approach accurately captures the complexity of their genetic and ecological differentiation, ensuring that future conservation strategies and taxonomic decisions remain biologically meaningful.

It is important to note here that despite its clear advantages, this modernized approach still faces practical challenges. For example, quantifying partial or low levels of gene flow remains methodologically complex, especially given that many widely accepted species exhibit limited gene flow. Further complications arise from distinguishing between historical and recent gene flow, which requires careful incorporation of temporal dimensions into analyses. Allopatric distributions also pose difficulties, as direct gene flow assessment is often impossible without laboratory conditions or inferred assumptions, necessitating alternative proxies such as morphological or ecological characteristics. Lastly, certain evolutionary scenarios—such as ring species, gradual clinal speciation, and paraphyletic speciation—complicate the delineation of distinct lineages, raising questions about whether the inclusive lineage must always be monophyletic.

#### 4.3.1 Description of *Melitaea diamina centranthi* ssp. nov

*Melitaea diamina centranthi* Sanjurjo‐Franch, Montiel‐Pantoja, Martínez‐Pérez, Spilani & Vila, 2025 ssp. nov.

ZooBank registration: 68698 AD1‐6610‐420A‐A86A‐88010CC8D0A0.

Type material: Table S1, Figure S8A–P.

Holotype, adult (Table S1, Figure S8A), SPAIN, Fuentes de Peñacorada, León, 973 m a.s.l., 42.829 latitudinal degrees, −5.1165 longitudinal degrees, 26.vi.2016, (I. Martínez); in coll. Institut de Biologia Evolutiva (CSIC‐UPF), Barcelona, Spain, under code RVcoll16L860, body stored in ethanol and wings as voucher (Figure S8A); COI GenBank Accession no.: MW500685.1.

Paratypes, SPAIN, 1 adult, Fuentes de Peñacorada, León, 4 adults Arroyo de la Boyariza, Geras, León, 1 adult, Arroyo Palanco, Geras, León, 3 adults, Vega de Gordón, León, 5 adults, Tarna, Asturias, 1 adult, Argovejo, León, 1 adult, Bejes, Cantabria; in coll. Institut de Biologia Evolutiva (CSIC‐Universitat Pompeu Fabra), Barcelona, Spain, body stored in ethanol and wings as voucher—bodies stored in ethanol and wings in vouchers (Figure S8B–P, Table S1).

Diagnosis: *Melitaea diamina centranthi* ssp. nov. can be distinguished from the other closely related subspecies on the basis of the mitochondrial DNA (in particular the sequence of the mitochondrial gene cytochrome c oxidase subunit I, COI). All sequenced individuals of *Melitaea diamina centranthi* ssp. nov. are differentiated unambiguously from all other sequenced allopatric *M*. diamina ssp. based on the following characters in the 658 bp COI barcode: guanine (G) versus adenine (A) in position 415; and G vs. A in position 553. In order to determine the DNA barcoding diagnostic characters for the subspecies described, in addition to sequences we generated in this study, we downloaded all publicly available sequences for *M*. *diamina*, which coincide with those illustrated in Dapporto et al. (2022).

The morphology of this taxon is also characteristic. The dorsal surface is pale tawny, noticeably less obscured than in the subspecies *M. diamina bustilloi* or *M. diamina diamina*, with the contrast particularly marked in the basal and submarginal areas of the forewing dorsum and across the entire dorsal surface of the hindwings. The basal discal mark on the forewing is bone‐shaped, with concave edges. In *M. diamina diamina*, the orange bands within the discal, postdiscal, and submarginal interspaces are typically veiled or barely discernible, whereas in *M. diamina centranthi* and *M. diamina codinai*, these areas remain more clearly defined and exhibit reduced dark pigmentation. Large marginal spots with straight edges are present, consistent with the pattern observed in other species of the genus and differing from that of the nominal subspecies. The ventral surface is lighter than in *M. diamina diamina*, with submarginal lunules on the hindwing showing a pale center surrounded by a darker hue.

Ecology: Scree slopes and limestone gorges hosting its host plant (*Centranthus lecoqii*) in shaded aspects or areas receiving limited sunlight.

Distribution: Central area of the Cantabrian Mountains, provinces of Asturias, Cantabria, and León.

Etymology: The subspecific name is based on the genus *Centranthus*, in reference to its exclusive association with the larval host plant *Centranthus lecoqii* Jord. (1852), a Mediterranean endemic. The choice of the subspecific name highlights that this is the first species of the genus *Centranthus* documented as a natural larval host for *Melitaea diamina*.

### 4.4 Conclusions

This study highlights the importance of adopting a nuanced taxonomic framework capable of accurately representing the complexity inherent to many biological lineages. By integrating genetic, ecological, and morphological data from Iberian populations of *M. diamina*, we have demonstrated that these lineages represent clear but incompletely separated evolutionary units. Rather than prematurely elevating each distinct population directly to species status—an approach that often oversimplifies biological complexity—we advocate for the recently modernized subspecies concept proposed by de Queiroz (2020). According to this updated definition, subspecies explicitly represent incompletely separated lineages nested within a more inclusive lineage, recognizing both lineage divergence and the occurrence of gene flow.

Conservation actions—and especially the funding allocated to them—are typically granted on the basis of species‐level taxonomy. However, there are notable exceptions. The IUCN allows for the assessment of extinction risk at the subspecies level, and within the Habitats Directive, among all populations of *Hesperia comma* , only the subspecies *catena* is listed. In the hope that the identification of subspecies will become as rigorous as the description of new species, we advocate for their recognition as legitimate targets for biodiversity conservation, increasingly integrated into both national and EU‐level strategies.

The populations of *M. diamina*, particularly those in the Iberian Peninsula such as the alarmingly declining Ports de Tortosa population, exemplify the need for such nuanced recognition. Embracing this updated concept of subspecies will not only foster greater biological accuracy and taxonomic stability but also provide policymakers with a meaningful classification framework, enabling targeted and effective conservation interventions for populations otherwise neglected by traditional species‐centric conservation strategies.

## Author Contributions

**Loukia Spilani:** conceptualization, formal analysis, investigation, visualization, writing – original draft, writing – review and editing. **Valéria Marques:** formal analysis, writing – review and editing. **Cecilia Montiel‐Pantoja:** conceptualization, resources, investigation, writing – original draft, writing – review and editing. **Miguel Sanjurjo‐Franch:** conceptualization, resources, investigation, writing – original draft, writing – review and editing. **Isabel Martínez‐Pérez:** conceptualization, resources, investigation, writing – original draft, writing – review and editing. **Sergio Montagud Alario:** conceptualization, resources, writing – review and editing. **Leonardo Dapporto:** resources, writing – review and editing. **Vlad Dincă:** resources, investigation, writing – review and editing. **Roger Vila:** conceptualization, resources, writing – original draft, writing – review and editing, project administration, supervision, funding acquisition.

## Funding

L.S. was supported by the Joan Oró grants program of the Department of Research and Universities of the Government of Catalonia (exp. 2024 FI‐3 00531) and the European Social Fund Plus, V.M. was supported by PRE2020‐094870 funded by MCIN/AEI/10.13039/501100011033 and “European Social Fund (ESF) Investing in your future”, V.D. was supported by the Research Council of Finland (Academy Research Fellow, grants no. 324988, 328895 and 352652) and R.V. was supported by grant PID2022‐139689NB‐I00 (MICIU/AEI/10.13039/501100011033 and ERDF, EU) and by grant 2021‐SGR‐00420 (Departament de Recerca i Universitats, Generalitat de Catalunya).

## Conflicts of Interest

The authors declare no conflicts of interest.

## Supporting information


**Figure S1:** Principal Component Analysis (PCA) of 40,096 SNPs excluding the single specimen from Kazakhstan. Colours and symbols correspond to the population groupings identified in the main analyses. (A) Visualization of PC1 (5.3%) and PC2 (4.1%). Excluding the Kazakh specimen reveals clearer separation among the European samples along PC2, particularly between the Central European (CEur) and Eastern European (EEur) groups, which was otherwise obscured in the full dataset visualization (Figure 2A). (B) Visualization of PC1 (5.3%) and PC3 (3.9%). Variance along PC3 is primarily driven by two specimens from Romania, which represent the easternmost samples in the dataset following the exclusion of the Kazakh specimen.
**Figure S2:** BIC‐based cluster detection for the Discriminant Analysis of Principal Components (DAPC). The Bayesian Information Criterion (BIC) was calculated for values of K ranging from 1 to 10. The optimal number of clusters (K = 4, indicated by the circled point) was selected at the lowest BIC value, reflecting the best‐supported partitioning of the dataset into genetically distinct groups.
**Figure S3:** ΔK plot for the global STRUCTURE analysis of 44,414 SNPs across K = 2–7. The ΔK method (Evanno et al. 2005) supports K = 2 as the primary clustering solution, corresponding to the split between the Iberian and Eurasian groups, with secondary peaks at K = 3 and K = 5 reflecting the hierarchical population structure further resolved by the analyses presented in Figure 3B,C.
**Figure S4:** Major clustering solutions recovered by the global STRUCTURE analysis of 44,414 SNPs for K = 3–6. Each vertical bar represents an individual, with colours indicating membership coefficients to each inferred cluster. Population groupings are indicated above, with subspecific designations in italics below. At K = 3 and K = 4, Pyrenean individuals are consistently recovered as admixed between the Iberian and Eurasian clusters. From K = 5 onwards, Pyrenean individuals are assigned their own distinct cluster, albeit retaining a degree of admixture. Wdiamina is resolved as a separate cluster from K = 4 onwards, while Wcodinai and Ecodinai remain grouped together across all values of K. At K = 6, the single specimen from Kazakhstan is recovered as a distinct cluster. Only the most frequently recovered clustering solution (major cluster) is shown for each value of K.
**Figure S5:** Concatenated maximum likelihood phylogeny of Melitaea diamina inferred by IQtree from a whole‐locus sequence alignment. Branch support values are shown at major nodes as ultrafast bootstrap support site concordance factors (sCF). Colours correspond to the population groupings identified in the main analyses, with subspecific designations in italics. The Kazakh specimen (Mdiam_RVcoll11K875_KZ) is recovered as basal to the EEurAsia clade with a notably longer branch, and Pyrenean samples are recovered as paraphyletic with respect to the rest of the Iberian clade, consistent with their admixed origin. *Melitaea protomedia* (Mprot_RVcoll11K088_CN) was used as the outgroup. Scale bar represents substitutions per site. Note the broken branch leading to the outgroup, indicating a substantially longer branch truncated for display purposes.
**Figure S6:** Species tree of Melitaea diamina inferred by ASTRAL from individual locus trees estimated by RAxML‐NG, and used as the starting topology for the PhyloNetworks analysis (Figure 5). Branch support values shown at major nodes represent multi‐locus bootstrap support (500 replicates) calculated from bootstrapped RAxML‐NG gene trees. Colours correspond to the population groupings identified in the main analyses, with subspecific designations in italics. Within the Iberian clade, Wcodinai and Ecodinai are recovered as sisters with full bootstrap support, with Wdiamina branching subsequently. The Pyrenean population is recovered as basal to the Iberian clade, though its monophyly reflects the a priori group assignment required by the analysis rather than a freely inferred topology. *Melitaea protomedia* was used as the outgroup. Scale bar represents coalescent units.
**Figure S7:** Wings of *Melitaea diamina bustilloi* ssp. reinst. specimens used in this study. Sample codes: (A) RVcoll18E600; (B) RVcoll18E610; (C) RVcoll19E309; (D) RVcoll19E310; (E) RVcoll19E311; (F) RVcoll19E314, (G) RVcoll19E319, (H) RVcoll19E330, (I) RVcoll20B630. Metadata in Table S1.
**Figure S8:** Wings of *Melitaea diamina centranthi* ssp. nov. (A) holotype from SPAIN, Fuentes de Peñacorada, León, 973 m a.s.l., 42.829 latitudinal degrees, −5.1165 longitudinal degrees, 26.vi.2016, (I. Martínez); in coll. Institut de Biologia Evolutiva (CSIC‐Universitat Pompeu Fabra), Barcelona, Spain, under code RVcoll16L860; (B‐P) paratypes with sample codes (B) RVcoll16L861, (C) RVcoll18E596, (D) RVcoll18E597, (E) RVcoll18E599, (F) RVcoll18E601, (G) RVcoll18E603, (H) RVcoll18E604, (I) RVcoll18E605, (J) RVcoll19E312, (K) RVcoll19E313, (L) RVcoll19E321, (M) RVcoll19E325, (N) RVcoll19E326, (O) RVcoll19E327, (P) RVcollB626. Metadata in Table S1.
**Figure S9:** Wings of *Melitaea diamina codinai* ssp. reinst. specimens used in this study. Sample codes: (A) 080211MY18; (B) 080211MY42; (C) 080211MY45. Metadata in Table S1.
**Figure S10:** Wings of *Melitaea diamina vernetensis* ssp. reinst. specimens used in this study Sample codes: (A) RVcoll07W139; (B) RVcoll07W140; (C) RVcoll08R115; (D) RVcoll09V317; (E) RVcoll17B592; (F) RVcoll17E128. Metadata in Table S1, Supporting Information.

**Table S1:** Table containing metadata regarding the specimens used in the current study.

## Data Availability

Raw ddRADseq FASTQ reads have been deposited in the NCBI BioProject database under accession PRJNA1337706. All downstream data and scripts are available on Figshare at DOI: 10.6084/m9.figshare.32542119.
