# Supplementary material for: A New Challenge to Species Delimitation: Remarkable Genomic and Ecological Diversity in the Butterfly Melitaea diamina
Source: Mol Ecol. 2026 Jul 22;35(14):e70474. doi: 10.1111/mec.70474 (PMC13389834; doi:10.1111/mec.70474)
Supplement: Supplementary file 1 — Figure S1: Principal Component Analysis (PCA) of 40,096 SNPs excluding the single specimen from Kazakhstan. Colours and symbols correspond to the population groupings identified in the main analyses. (A) Visualization of PC1 (5.3%) and PC2 (4.1%). Excluding the Kazakh specimen reveals clearer separation among the European samples along PC2, particularly between the Central European (CEur) and Eastern European (EEur) groups, which was otherwise obscured in the full dataset visualization (Figure 2A). (B) Visualization of PC1 (5.3%) and PC3 (3.9%). Variance along PC3 is primarily driven by two specimens from Romania, which represent the easternmost samples in the dataset following the exclusion of the Kazakh specimen. Figure S2: BIC‐based cluster detection for the Discriminant Analysis of Principal Components (DAPC). The Bayesian Information Criterion (BIC) was calculated for values of K ranging from 1 to 10. The optimal number of clusters (K = 4, indicated by the circled point) was selected at the lowest BIC value, reflecting the best‐supported partitioning of the dataset into genetically distinct groups. Figure S3: ΔK plot for the global STRUCTURE analysis of 44,414 SNPs across K = 2–7. The ΔK method (Evanno et al. 2005) supports K = 2 as the primary clustering solution, corresponding to the split between the Iberian and Eurasian groups, with secondary peaks at K = 3 and K = 5 reflecting the hierarchical population structure further resolved by the analyses presented in Figure 3B,C. Figure S4: Major clustering solutions recovered by the global STRUCTURE analysis of 44,414 SNPs for K = 3–6. Each vertical bar represents an individual, with colours indicating membership coefficients to each inferred cluster. Population groupings are indicated above, with subspecific designations in italics below. At K = 3 and K = 4, Pyrenean individuals are consistently recovered as admixed between the Iberian and Eurasian clusters. From K = 5 onwards, Pyrenean individuals are assigned [file MEC-35-e70474-s001.docx]

**Supplemental Information for:**

**A new challenge to species delimitation: Remarkable genomic diversity and ecological divergence in the butterfly *Melitaea diamina***

Loukia Spilani, Valéria Marques, Cecilia Montiel-Pantoja, Miguel Sanjurjo-Franch, Isabel Martínez-Pérez, Sergio Montagud Alario, Leonardo Dapporto, Vlad Dincă, Roger Vila





**Figure S1:** Principal Component Analysis (PCA) of 40,096 SNPs excluding the single specimen from Kazakhstan. Colours and symbols correspond to the population groupings identified in the main analyses. (A) Visualisation of PC1 (5.3%) and PC2 (4.1%). Excluding the Kazakh specimen reveals clearer separation among the European samples along PC2, particularly between the Central European (CEur) and Eastern European (EEur) groups, which was otherwise obscured in the full dataset visualisation (Fig. 2A). (B) Visualisation of PC1 (5.3%) and PC3 (3.9%). Variance along PC3 is primarily driven by two specimens from Romania, which represent the easternmost samples in the dataset following the exclusion of the Kazakh specimen.


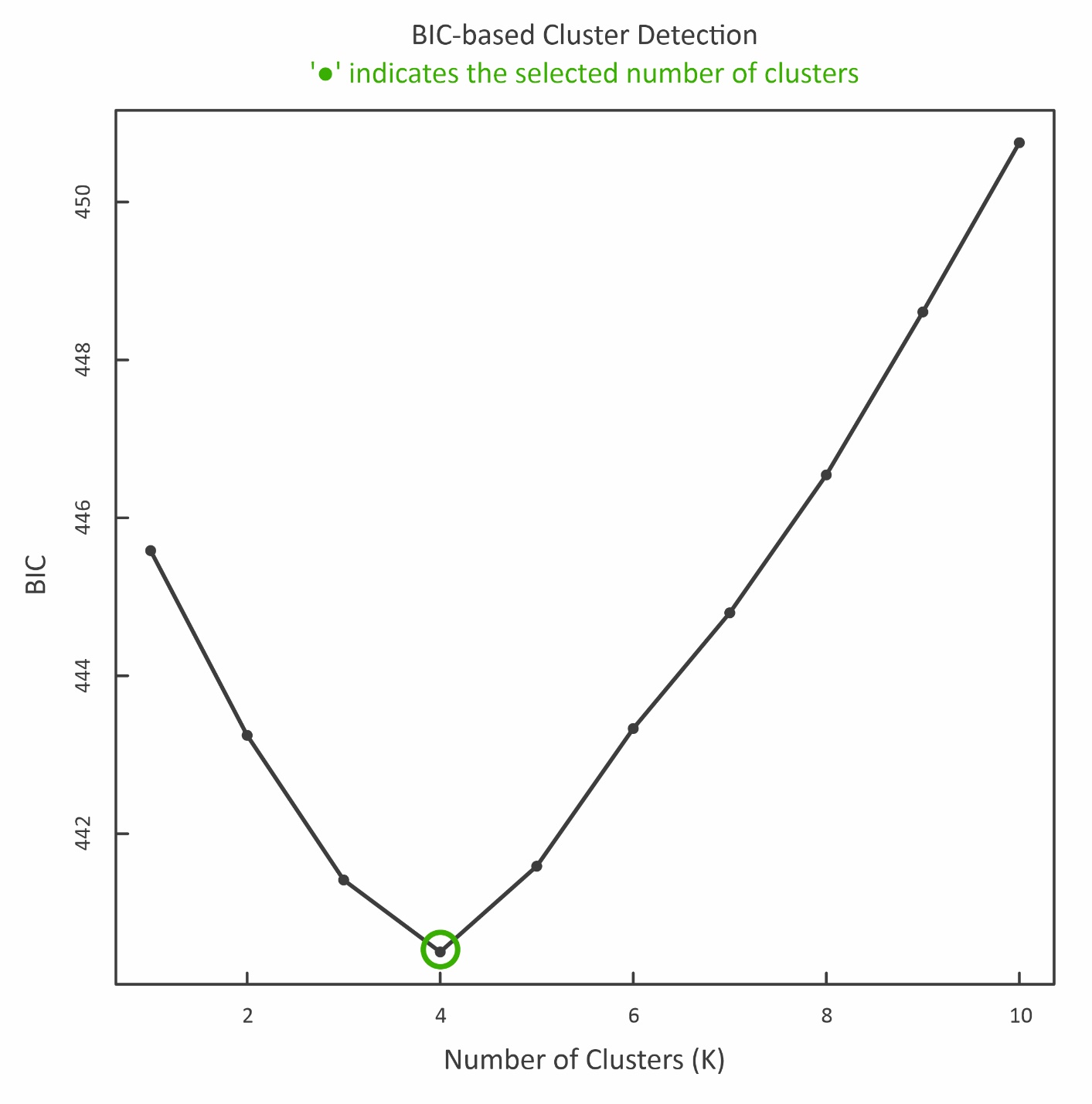


**Figure S2:** BIC-based cluster detection for the Discriminant Analysis of Principal Components (DAPC). The Bayesian Information Criterion (BIC) was calculated for values of K ranging from 1 to 10. The optimal number of clusters (K = 4, indicated by the circled point) was selected at the lowest BIC value, reflecting the best-supported partitioning of the dataset into genetically distinct groups.


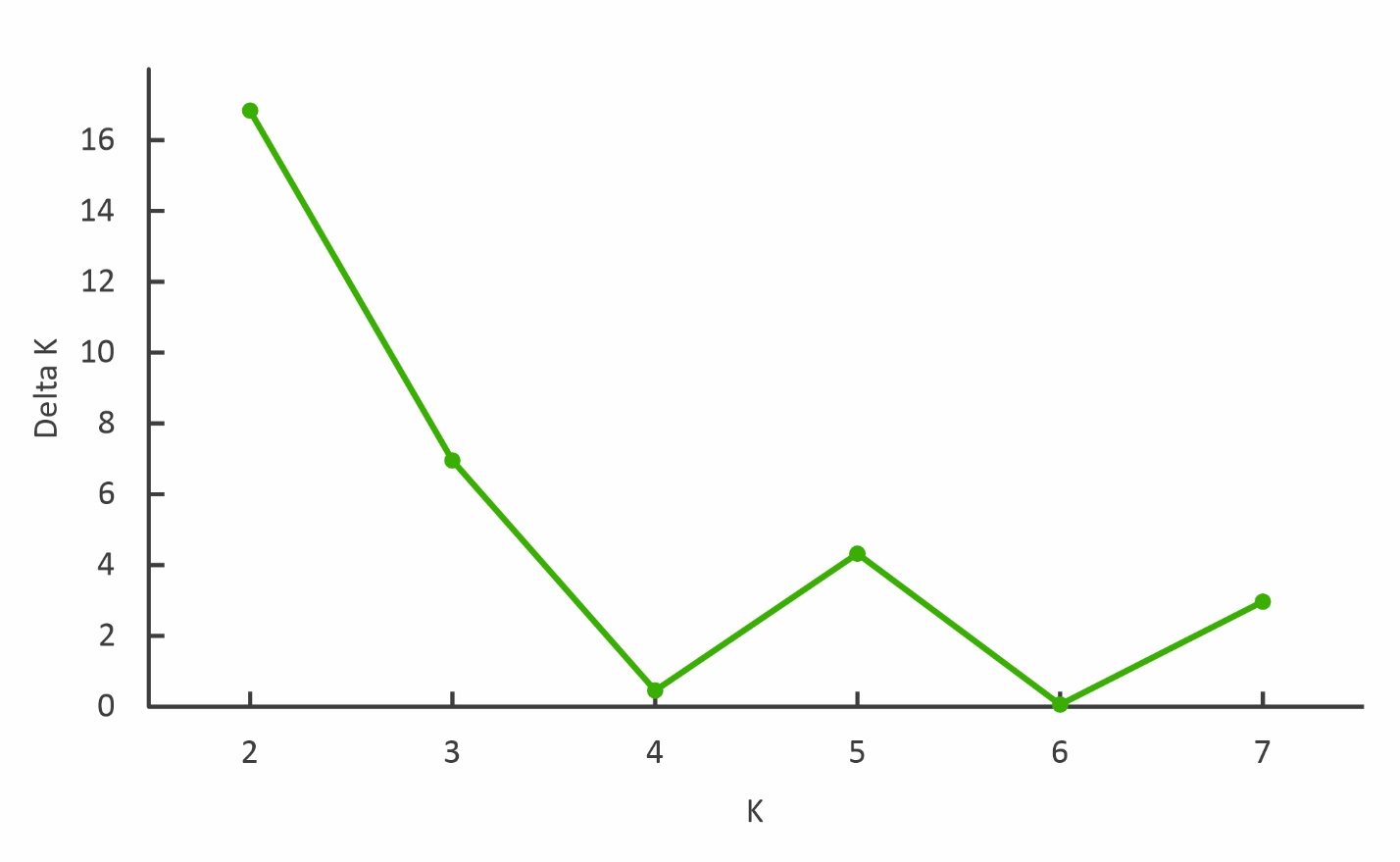


**Figure S3:** ΔK plot for the global STRUCTURE analysis of 44,414 SNPs across K = 2–7. The ΔK method (Evanno et al. 2005) supports K = 2 as the primary clustering solution, corresponding to the split between the Iberian and Eurasian groups, with secondary peaks at K = 3 and K = 5 reflecting the hierarchical population structure further resolved by the analyses presented in Figures 3B and 3C.


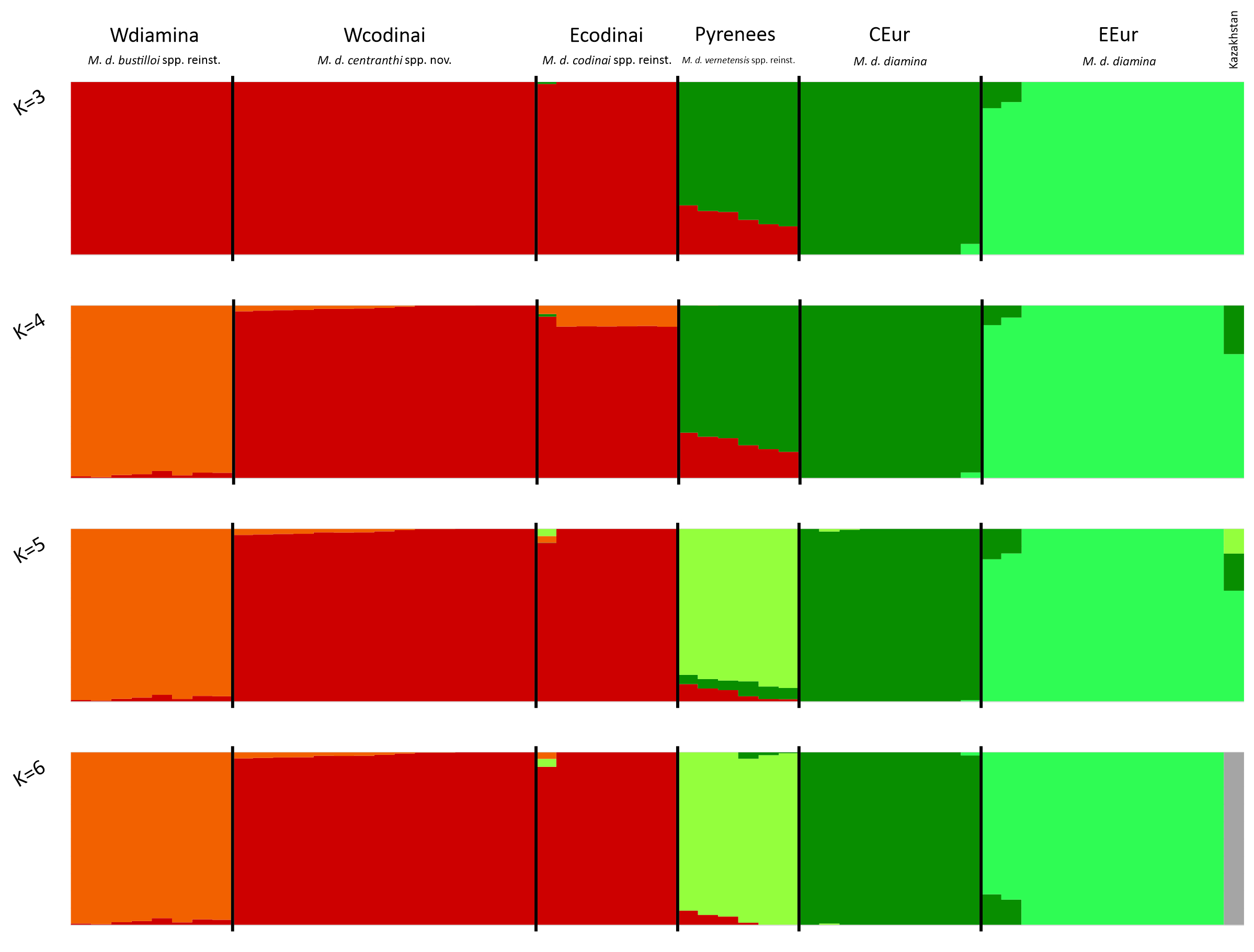


**Figure S4:** Major clustering solutions recovered by the global STRUCTURE analysis of 44,414 SNPs for K = 3–6. Each vertical bar represents an individual, with colours indicating membership coefficients to each inferred cluster. Population groupings are indicated above, with subspecific designations in italics below. At K = 3 and K = 4, Pyrenean individuals are consistently recovered as admixed between the Iberian and Eurasian clusters. From K = 5 onwards, Pyrenean individuals are assigned their own distinct cluster, albeit retaining a degree of admixture. Wdiamina is resolved as a separate cluster from K = 4 onwards, while Wcodinai and Ecodinai remain grouped together across all values of K. At K = 6, the single specimen from Kazakhstan is recovered as a distinct cluster. Only the most frequently recovered clustering solution (major cluster) is shown for each value of K.


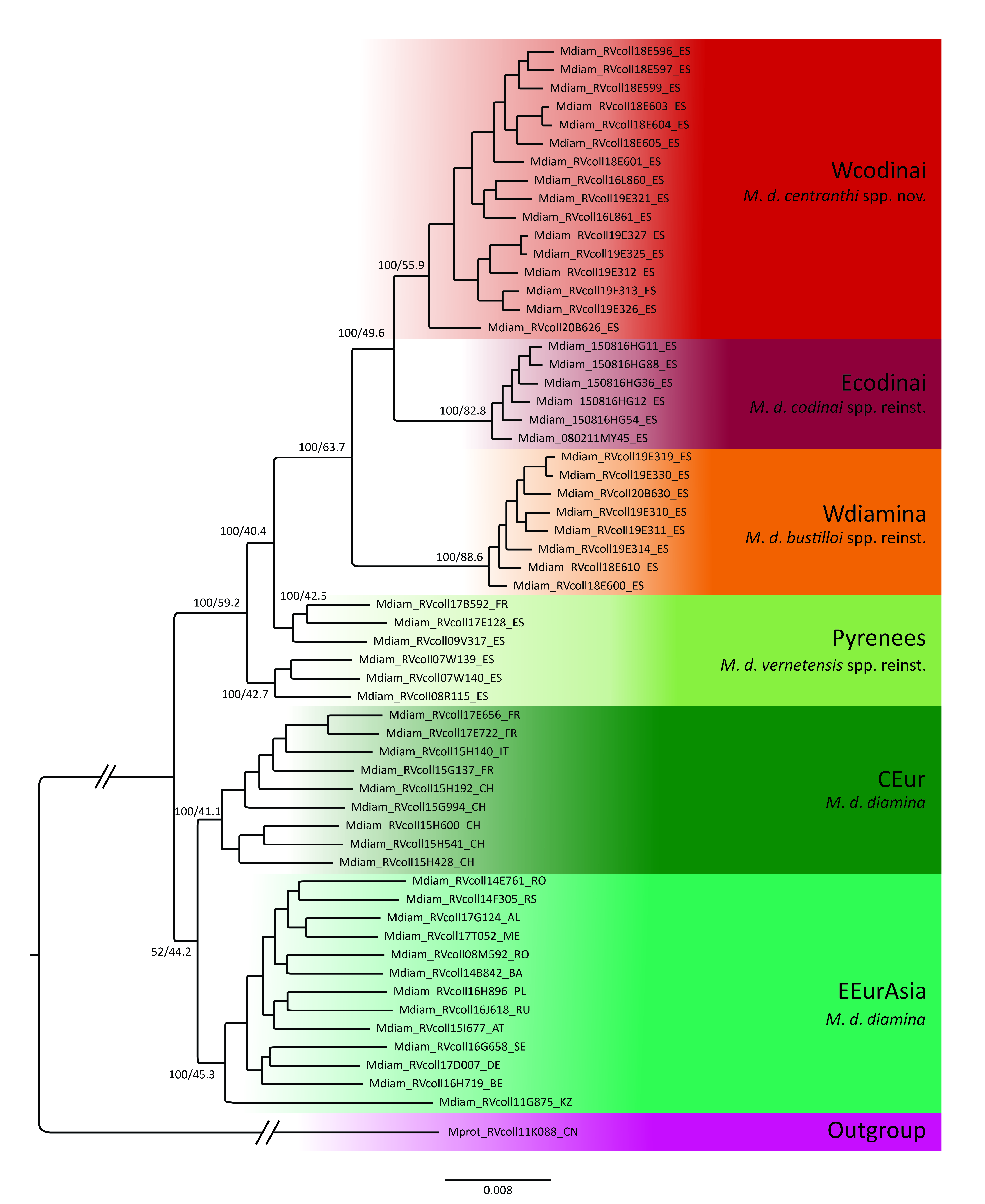


**Figure S5:** Concatenated maximum likelihood phylogeny of Melitaea diamina inferred by IQtree from a whole-locus sequence alignment. Branch support values are shown at major nodes as ultrafast bootstrap support site concordance factors (sCF). Colours correspond to the population groupings identified in the main analyses, with subspecific designations in italics. The Kazakh specimen (Mdiam_RVcoll11K875_KZ) is recovered as basal to the EEurAsia clade with a notably longer branch, and Pyrenean samples are recovered as paraphyletic with respect to the rest of the Iberian clade, consistent with their admixed origin. Melitaea protomedia (Mprot_RVcoll11K088_CN) was used as the outgroup. Scale bar represents substitutions per site. Note the broken branch leading to the outgroup, indicating a substantially longer branch truncated for display purposes.

**Figure S6:** Species tree of Melitaea diamina inferred by ASTRAL from individual locus trees estimated by RAxML-NG, and used as the starting topology for the PhyloNetworks analysis (Fig. 5). Branch support values shown at major nodes represent multi-locus bootstrap support (500 replicates) calculated from bootstrapped RAxML-NG gene trees. Colours correspond to the population groupings identified in the main analyses, with subspecific designations in italics. Within the Iberian clade, Wcodinai and Ecodinai are recovered as sisters with full bootstrap support, with Wdiamina branching subsequently. The Pyrenean population is recovered as basal to the Iberian clade, though its monophyly reflects the a priori group assignment required by the analysis rather than a freely inferred topology. Melitaea protomedia was used as the outgroup. Scale bar represents coalescent units.


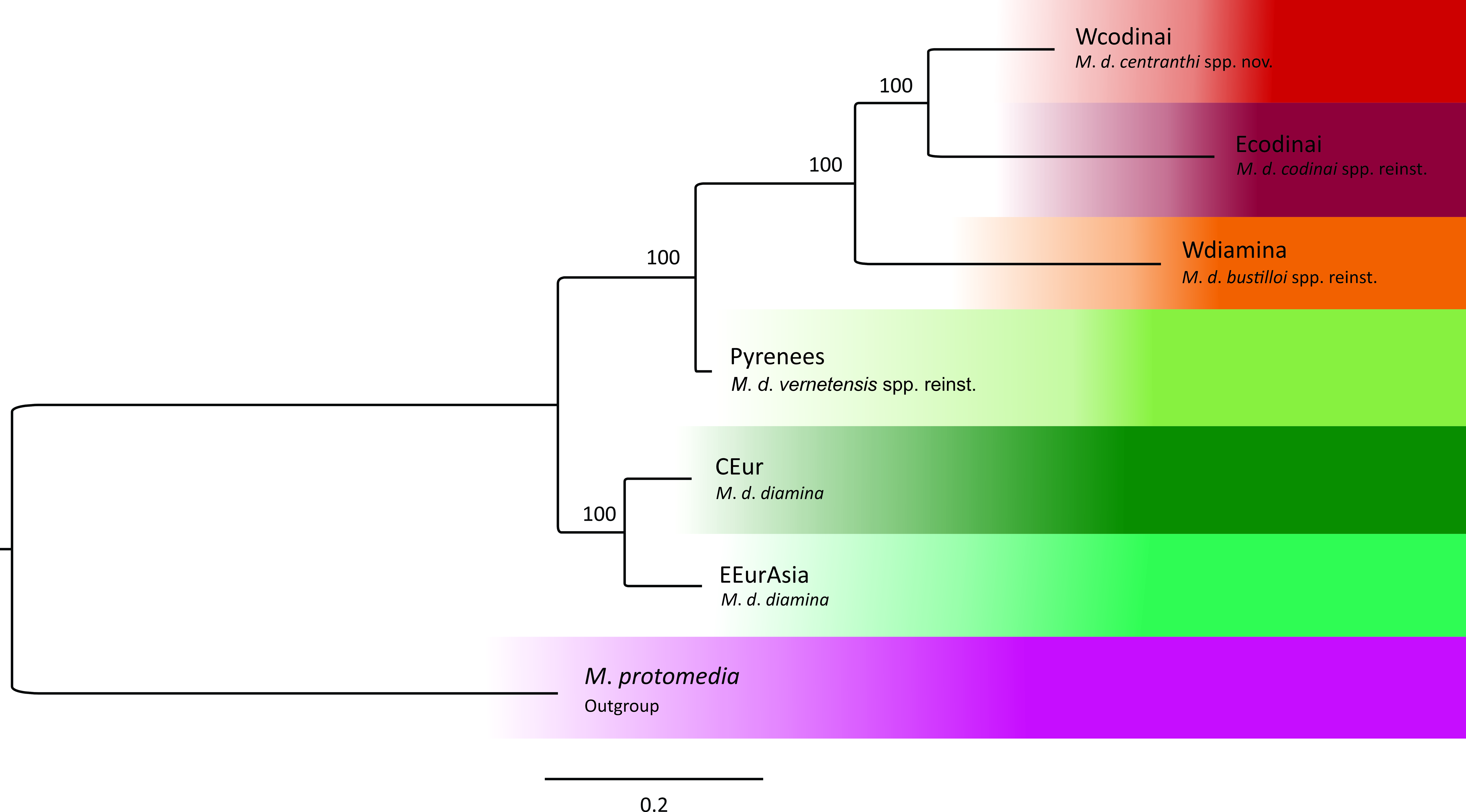

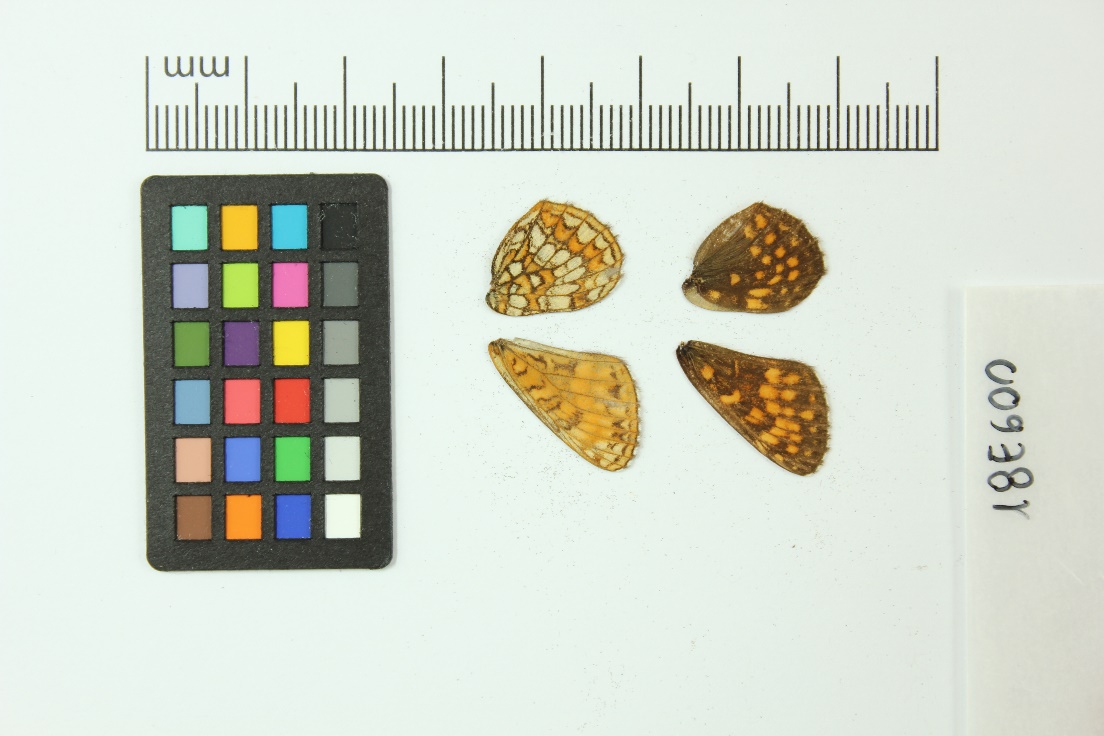


**A**


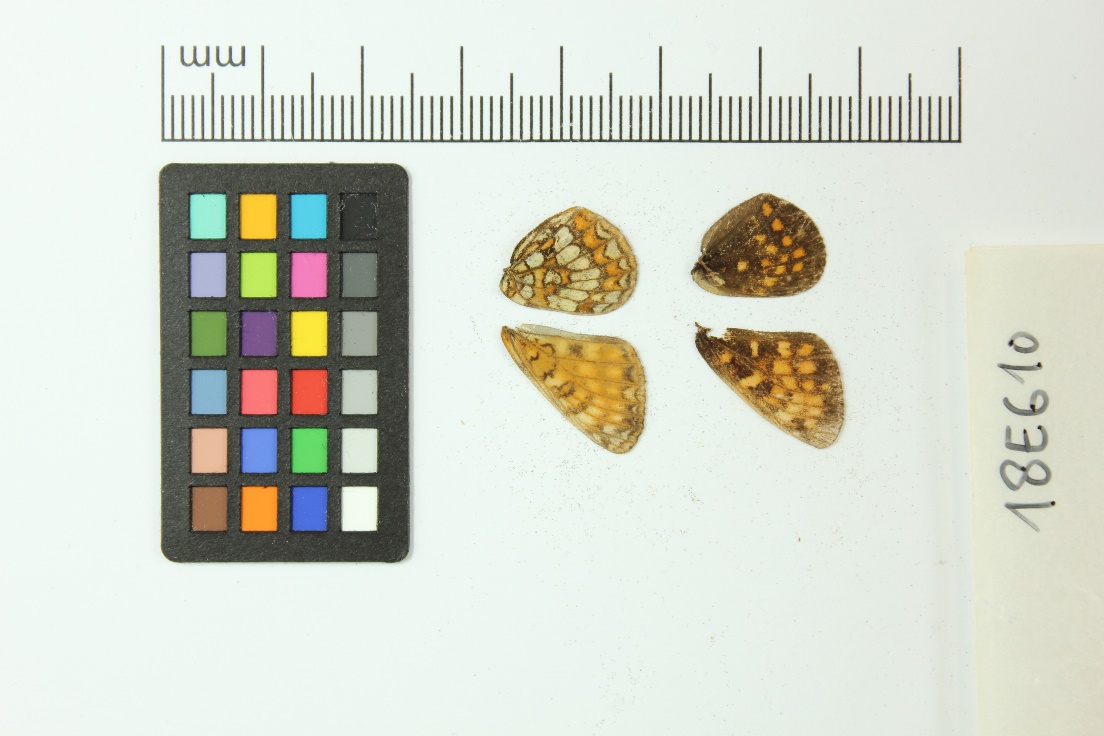


**B**


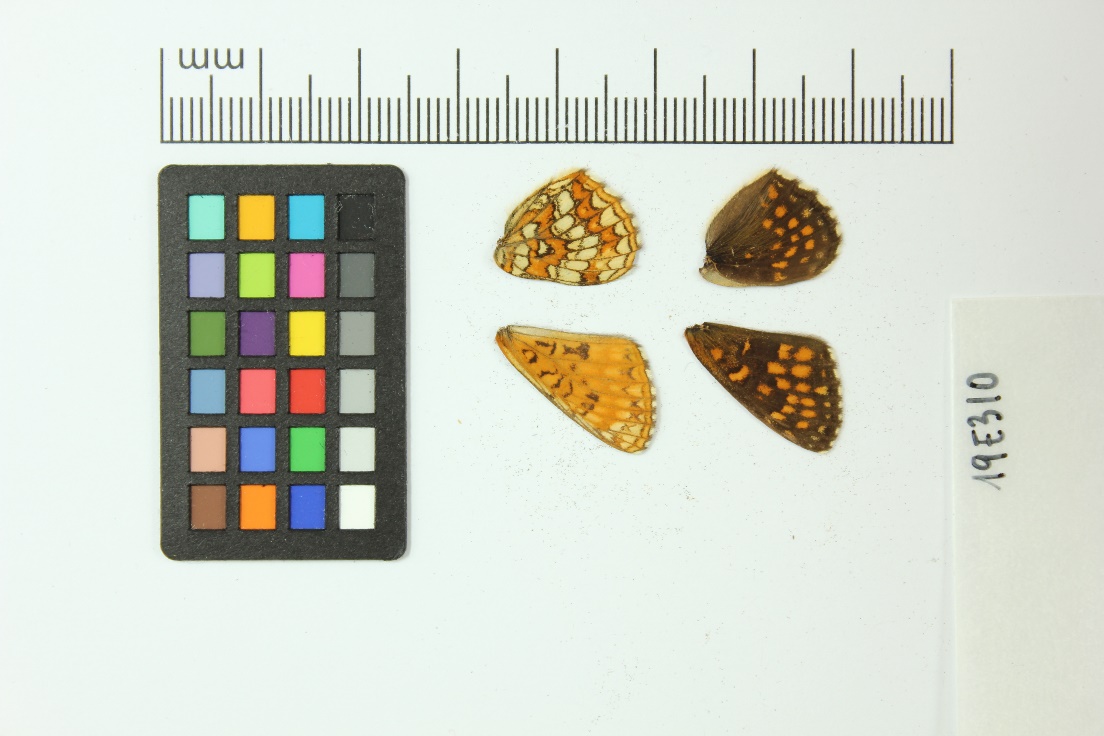


**D**


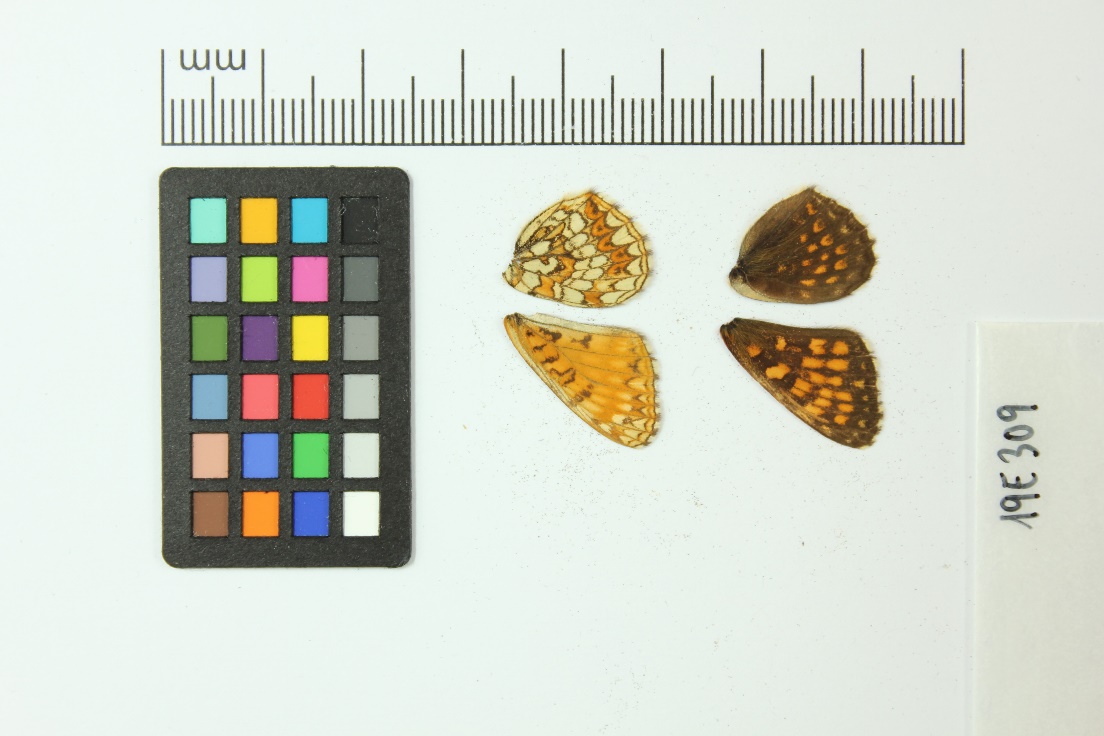


**C**


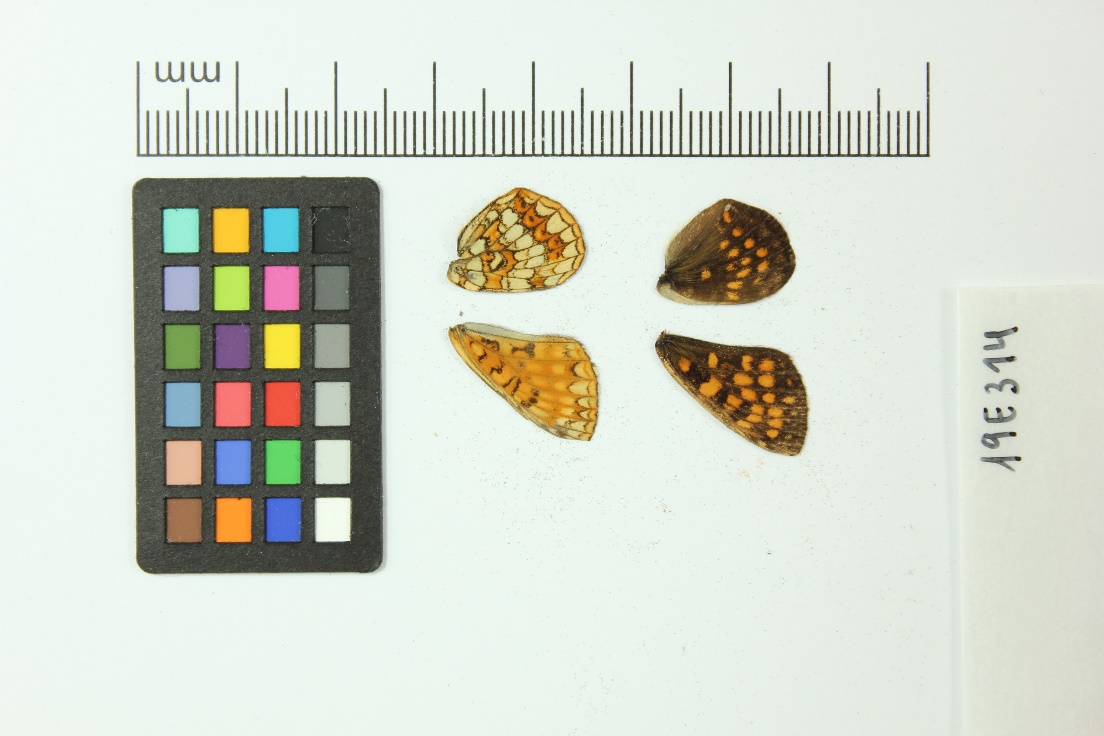


**F**


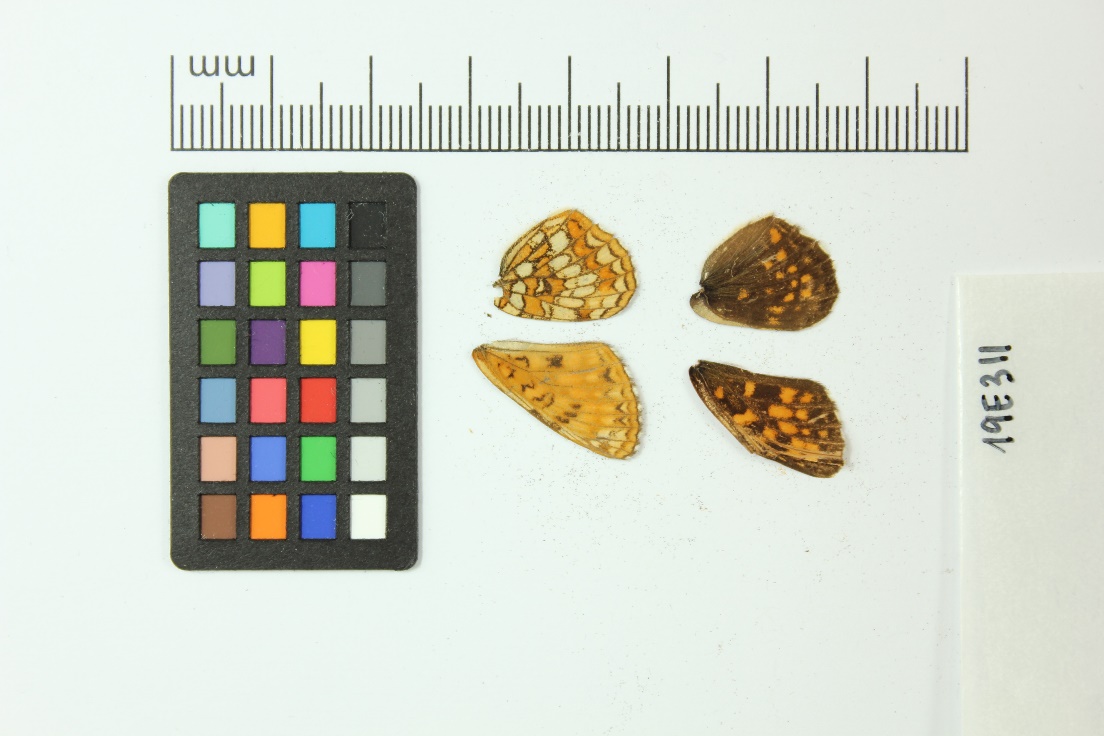


**E**


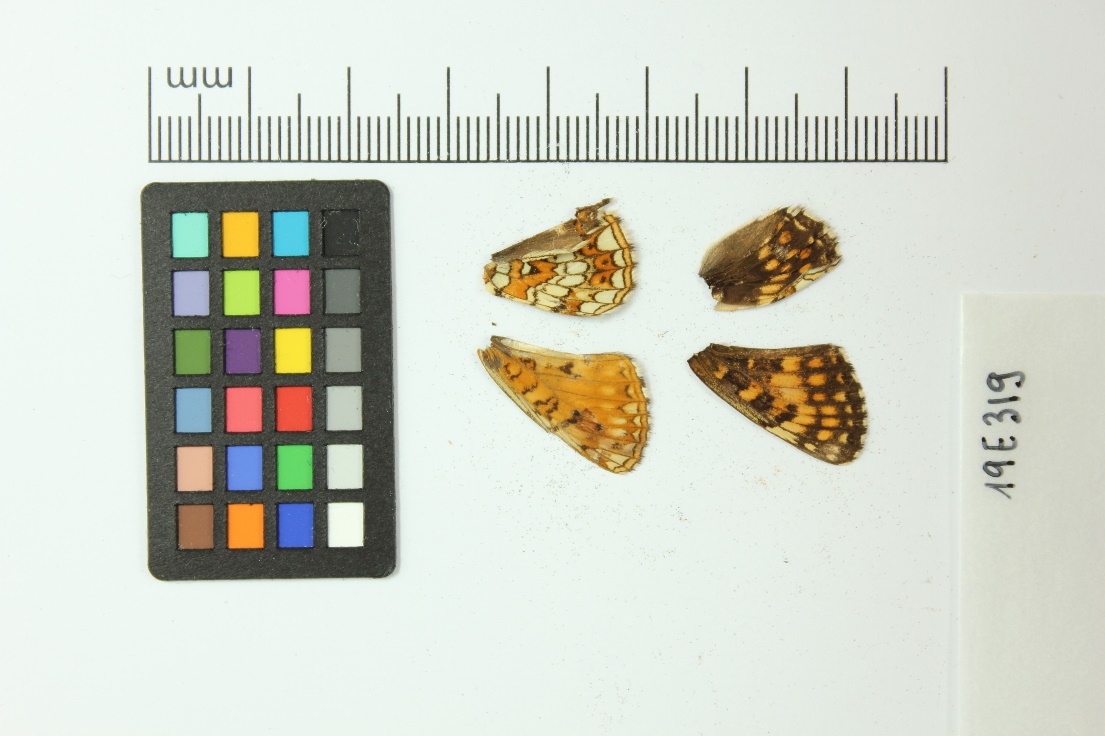


**G**


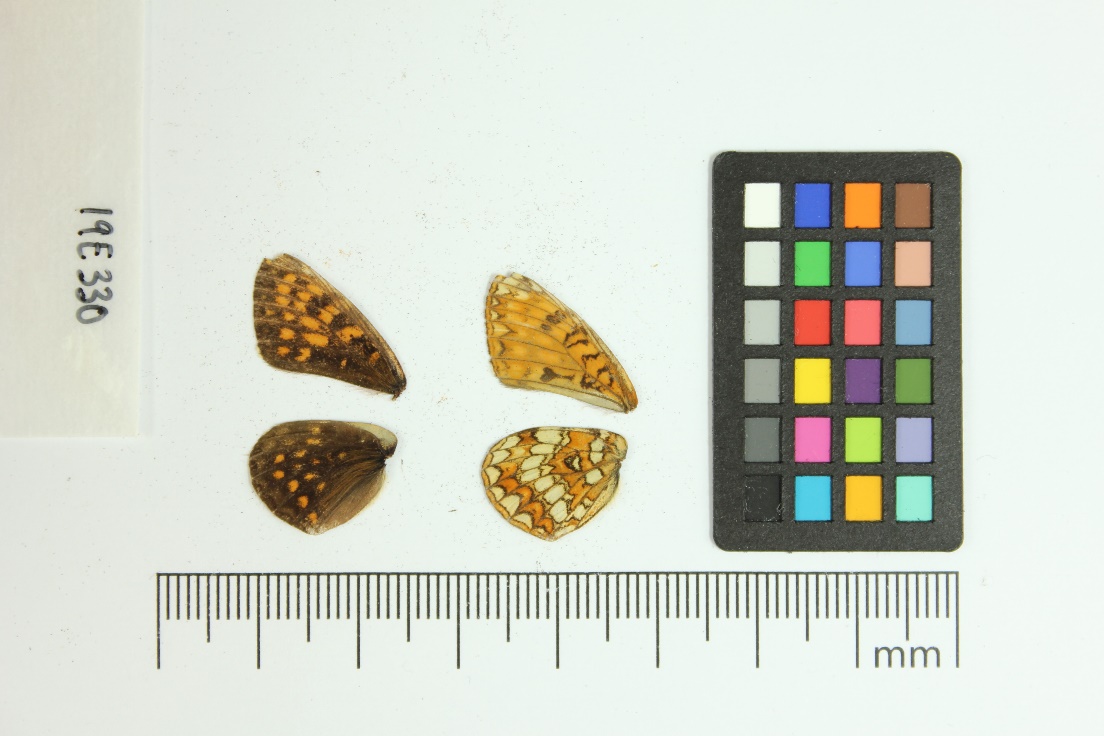


**H**


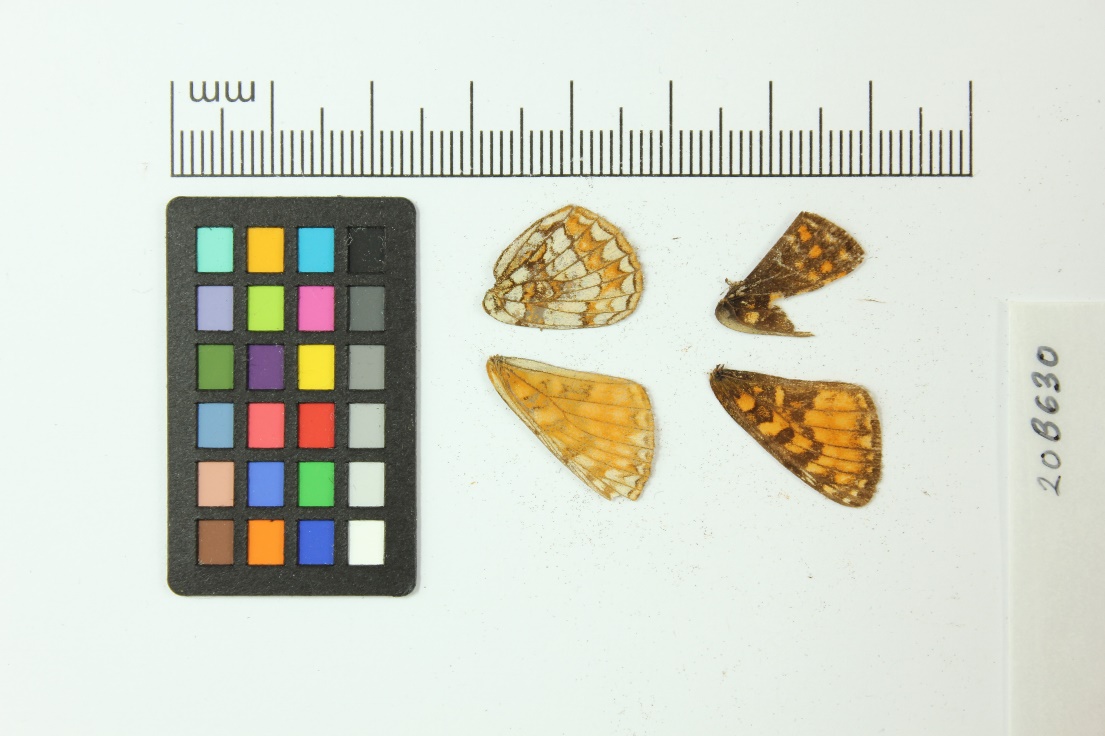


**I**

**Figure S7:** Wings of Melitaea diamina bustilloi ssp. reinst. specimens used in this study. Sample codes: **(A)** RVcoll18E600; **(B)** RVcoll18E610; **(C)** RVcoll19E309; **(D)** RVcoll19E310; **(E)** RVcoll19E311; **(F)** RVcoll19E314, **(G)** RVcoll19E319, **(H)** RVcoll19E330, **(I)** RVcoll20B630. Metadata in Table S1.


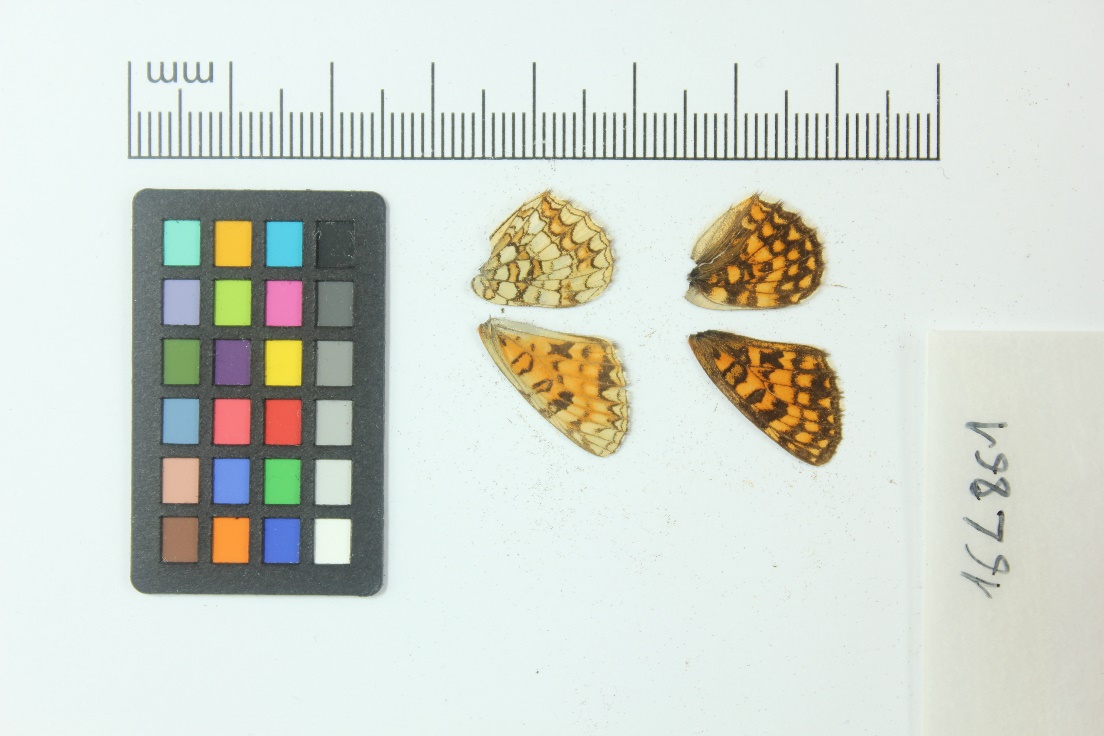


**B (paratype)**


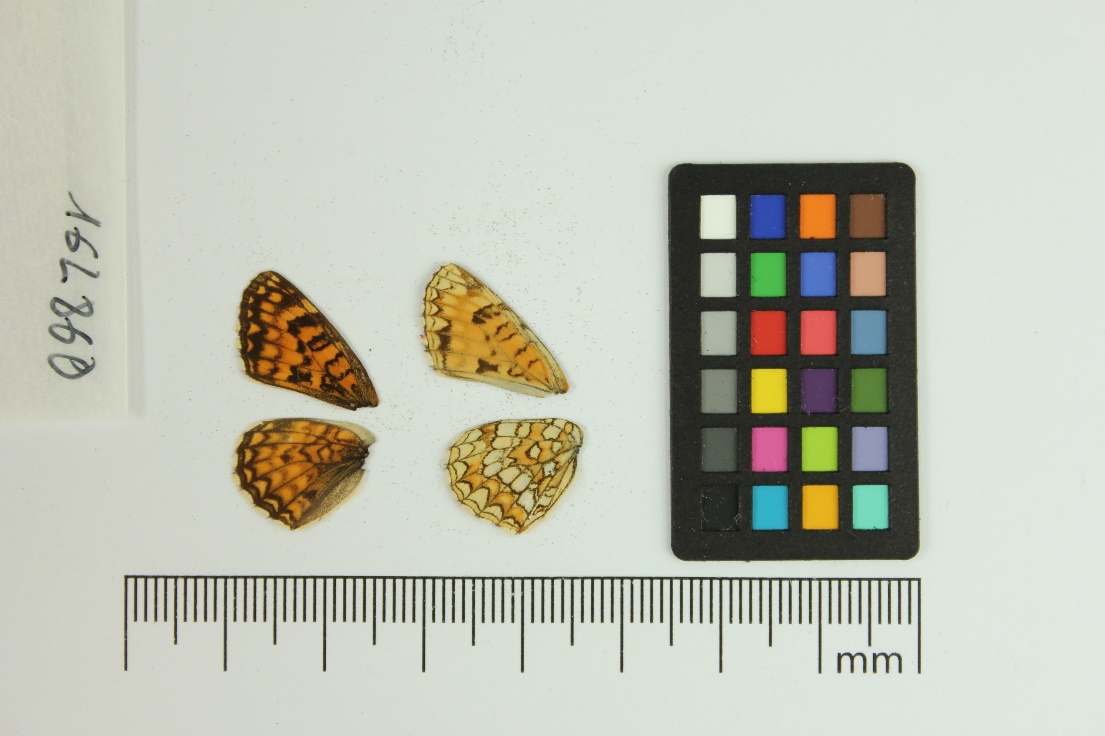


**A (holotype)**

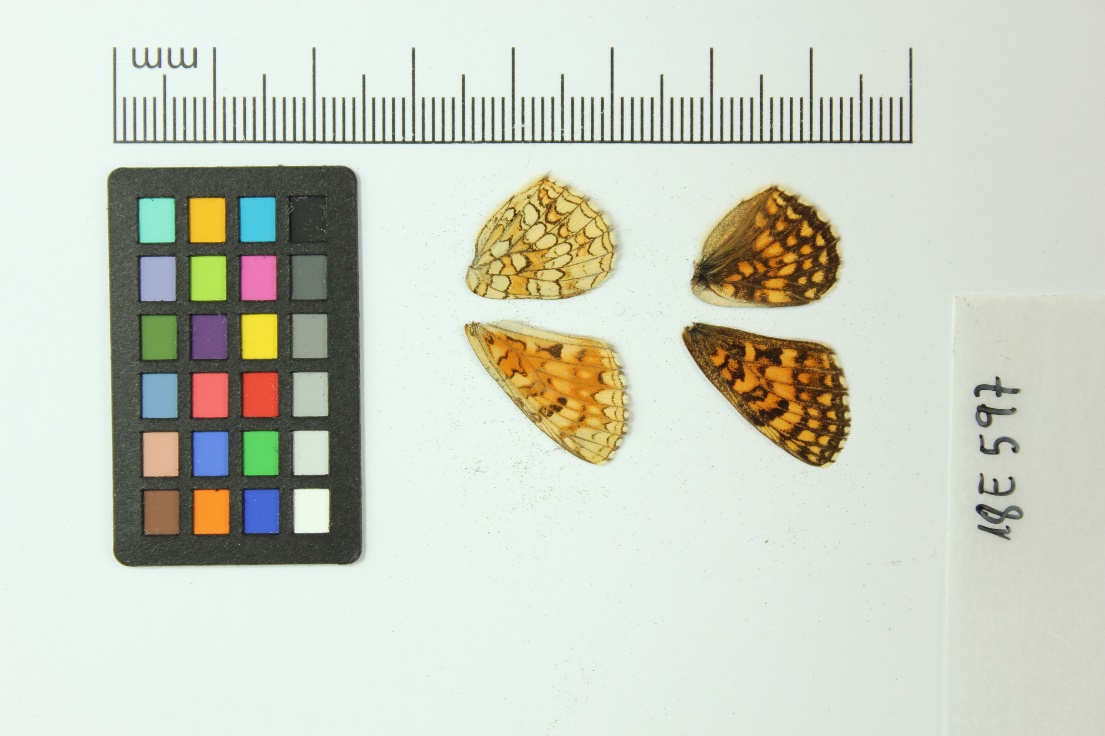


**D (paratype)**


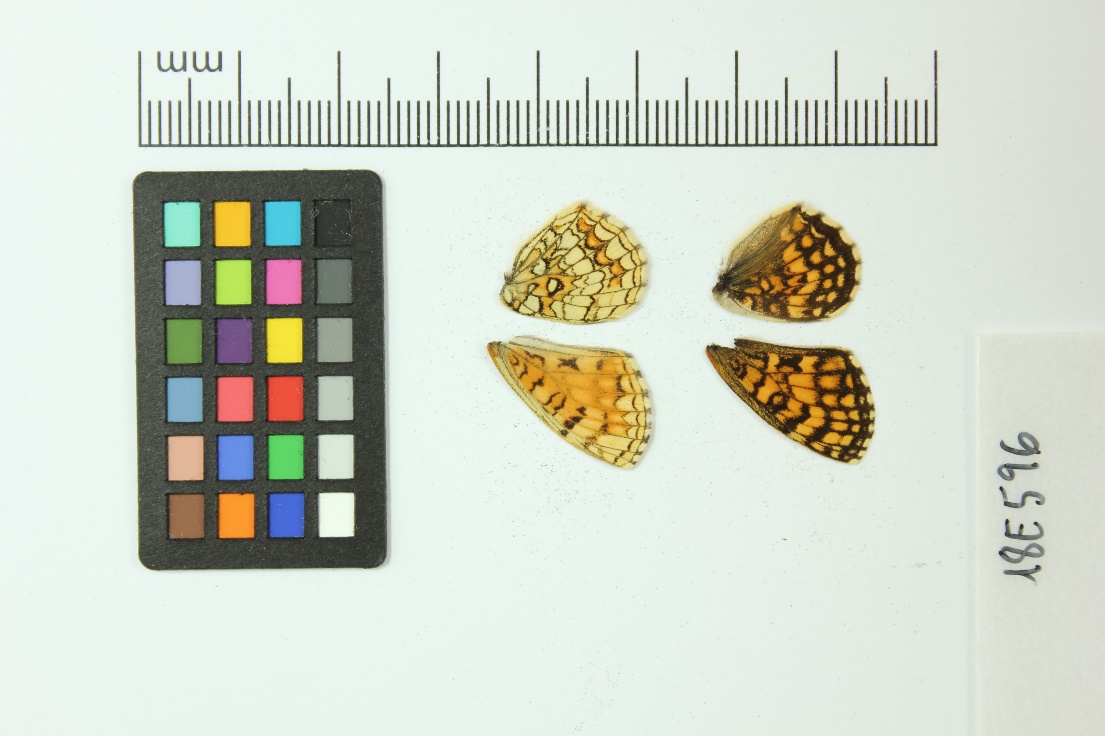


**C (paratype)**


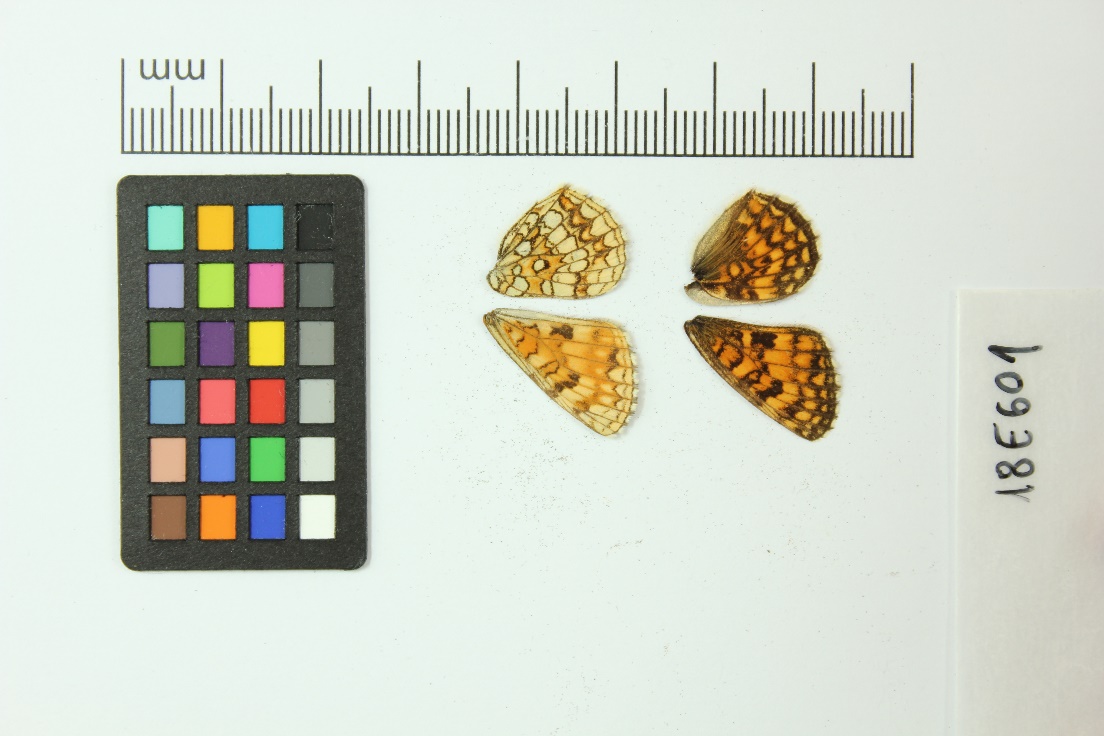


**F (paratype)**


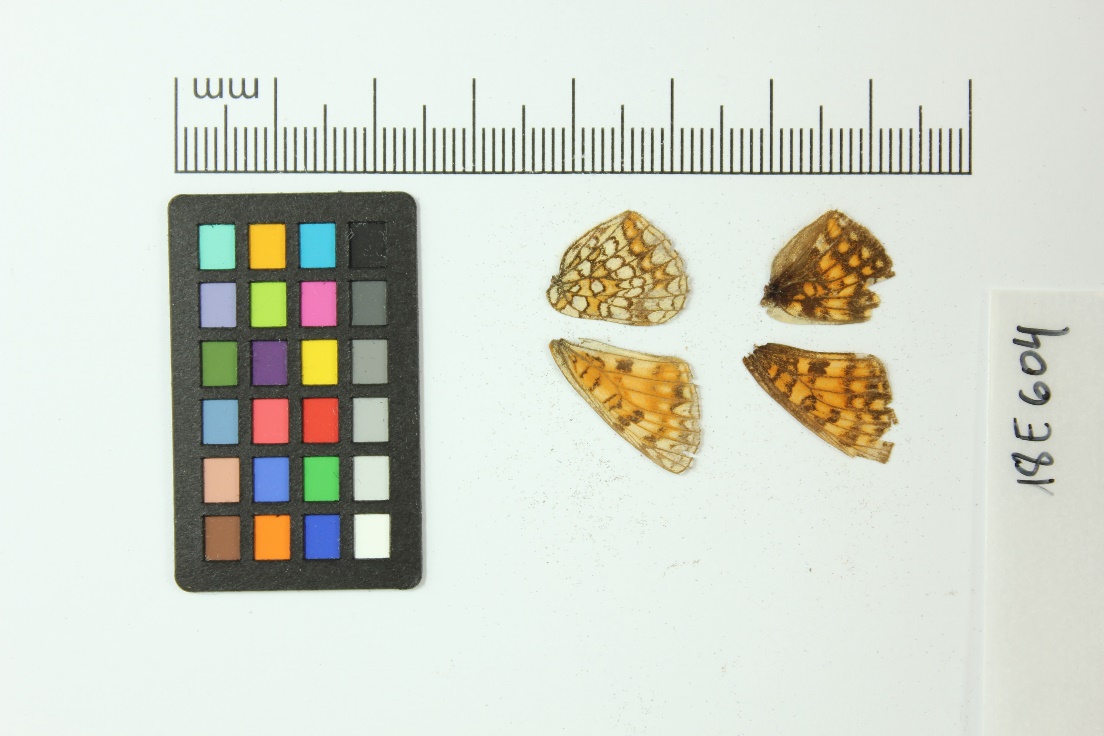


**H (paratype)**


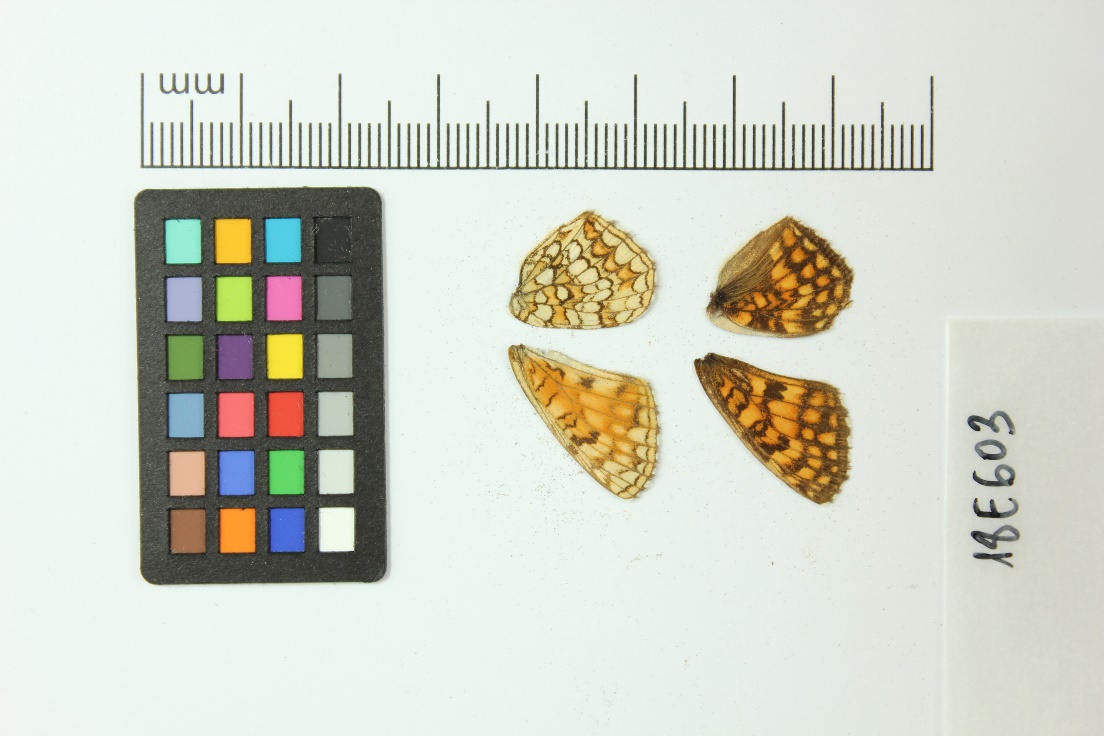


**G (paratype)**


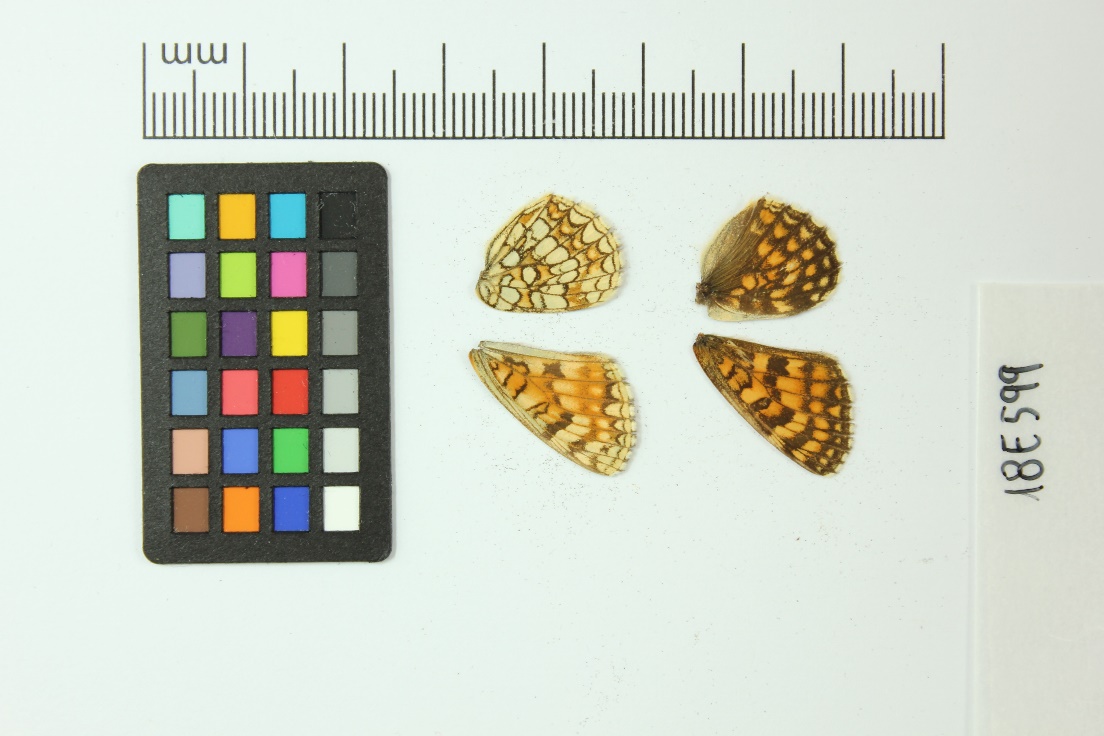


**E (paratype)**

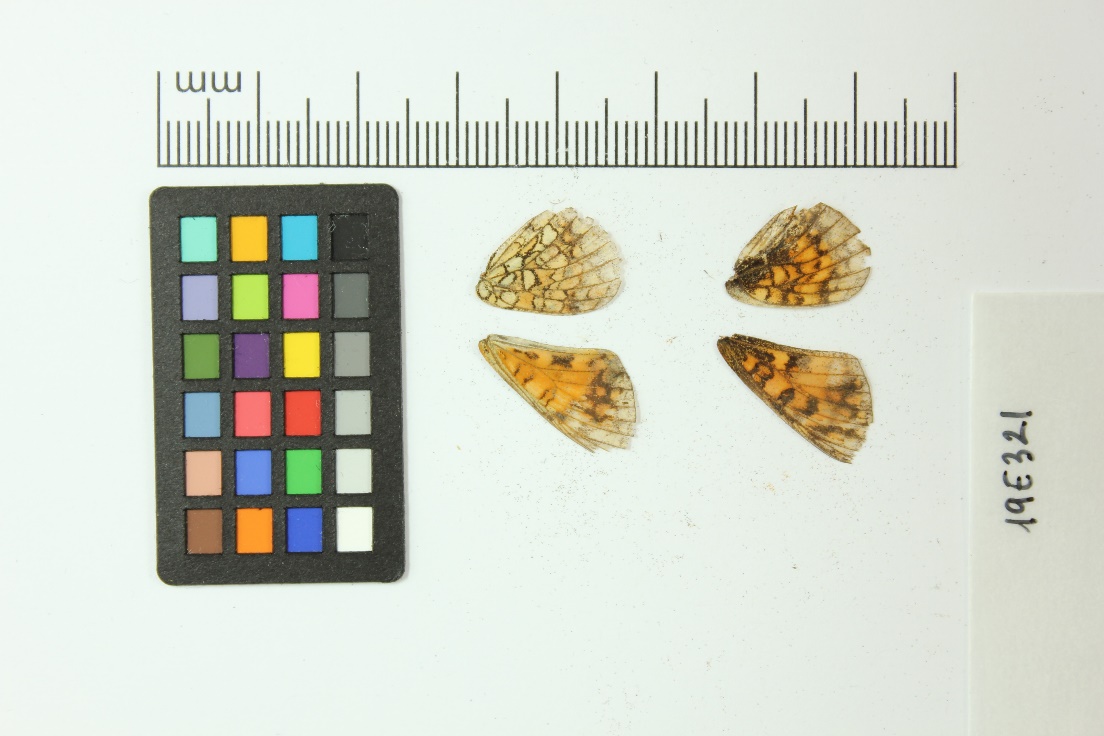


**L (paratype)**

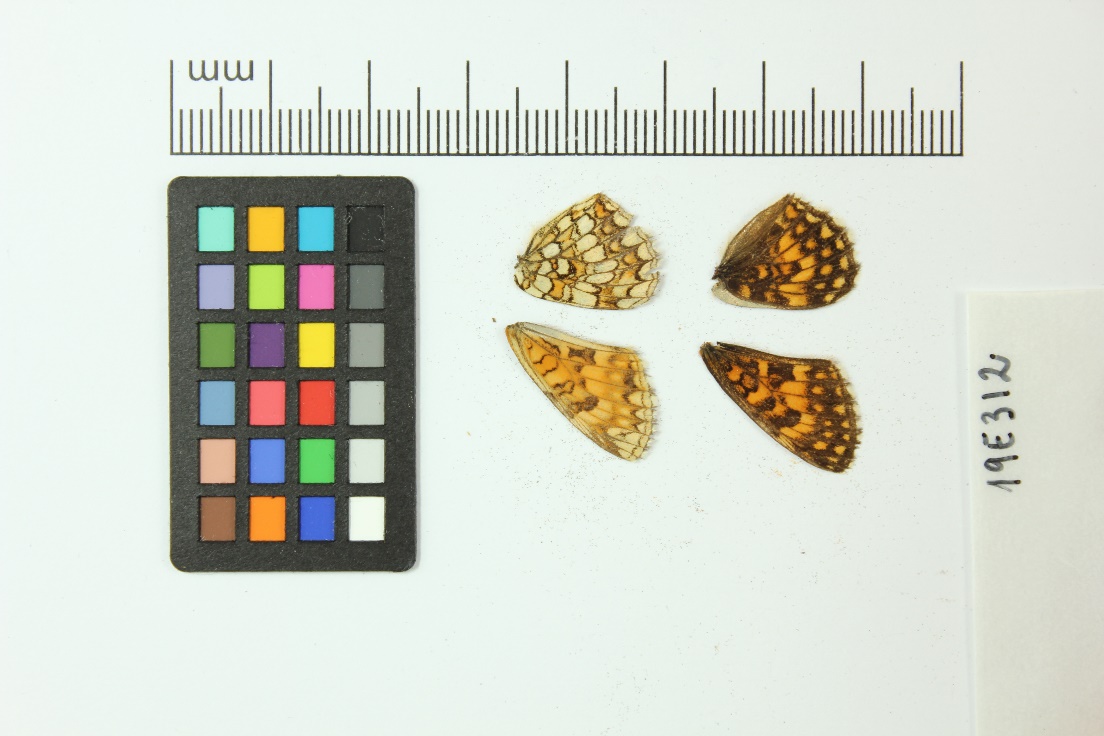


**J (paratype)**


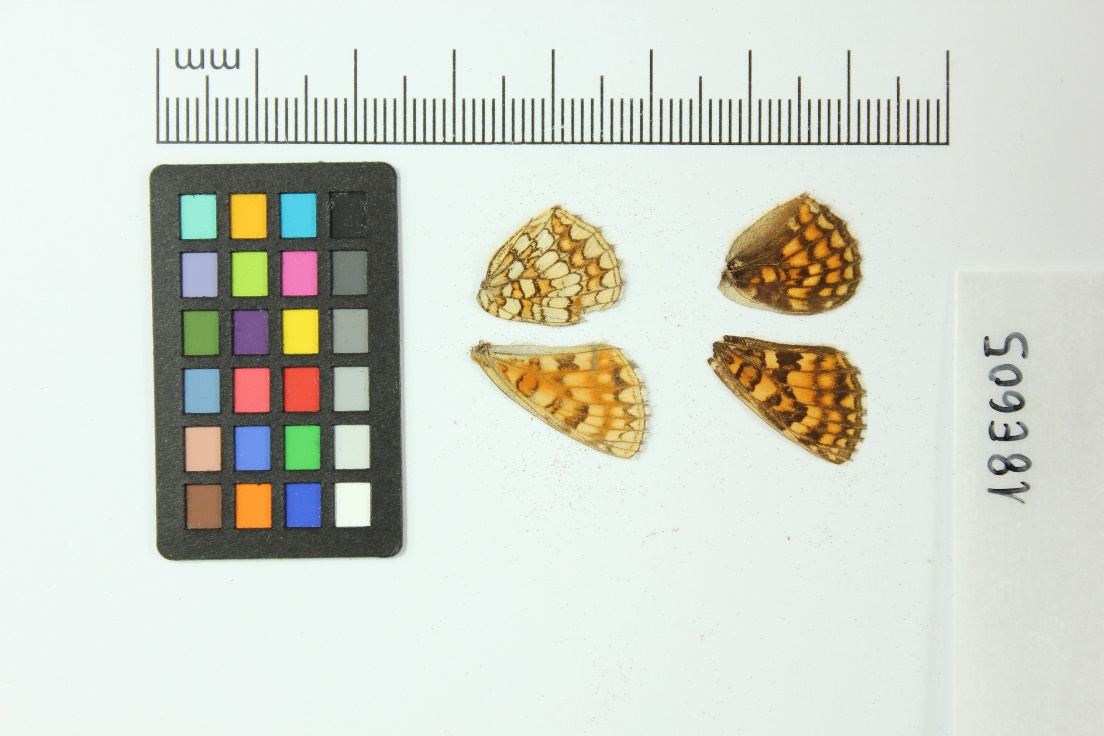


**I (paratype)**


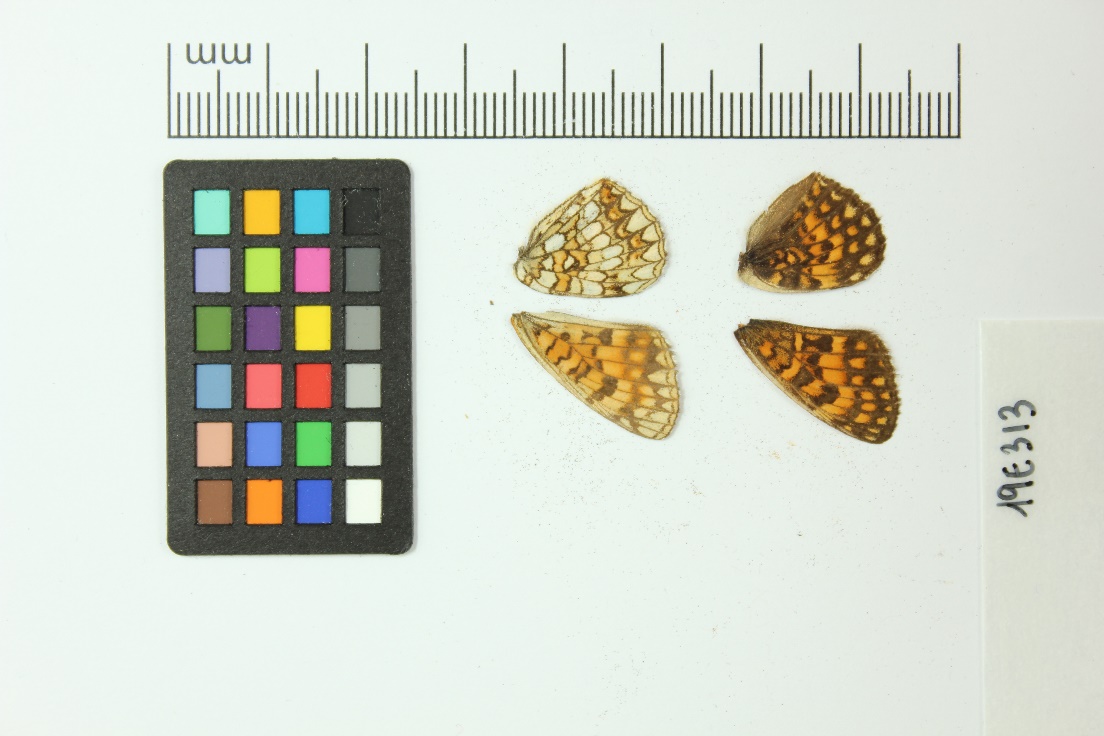


**K (paratype)**

**Figure S8:** Wings of Melitaea diamina centranthi ssp. nov. **(A)** holotype from SPAIN, Fuentes de Peñacorada, León, 973 m a.s.l., 42.829 latitudinal degrees, -5.1165 longitudinal degrees, 26.vi.2016, (I. Martínez); in coll. Institut de Biologia Evolutiva (CSIC-Universitat Pompeu Fabra), Barcelona, Spain, under code RVcoll16L860; (B-P) paratypes with sample codes **(B)** RVcoll16L861, **(C)** RVcoll18E596, **(D)** RVcoll18E597, **(E)** RVcoll18E599, **(F)** RVcoll18E601, **(G)** RVcoll18E603, **(H)** RVcoll18E604, **(I)** RVcoll18E605, **(J)** RVcoll19E312, **(K**) RVcoll19E313, **(L)** RVcoll19E321, **(M)** RVcoll19E325, **(N)** RVcoll19E326, **(O)** RVcoll19E327, **(P)** RVcollB626. Metadata in Table S1.


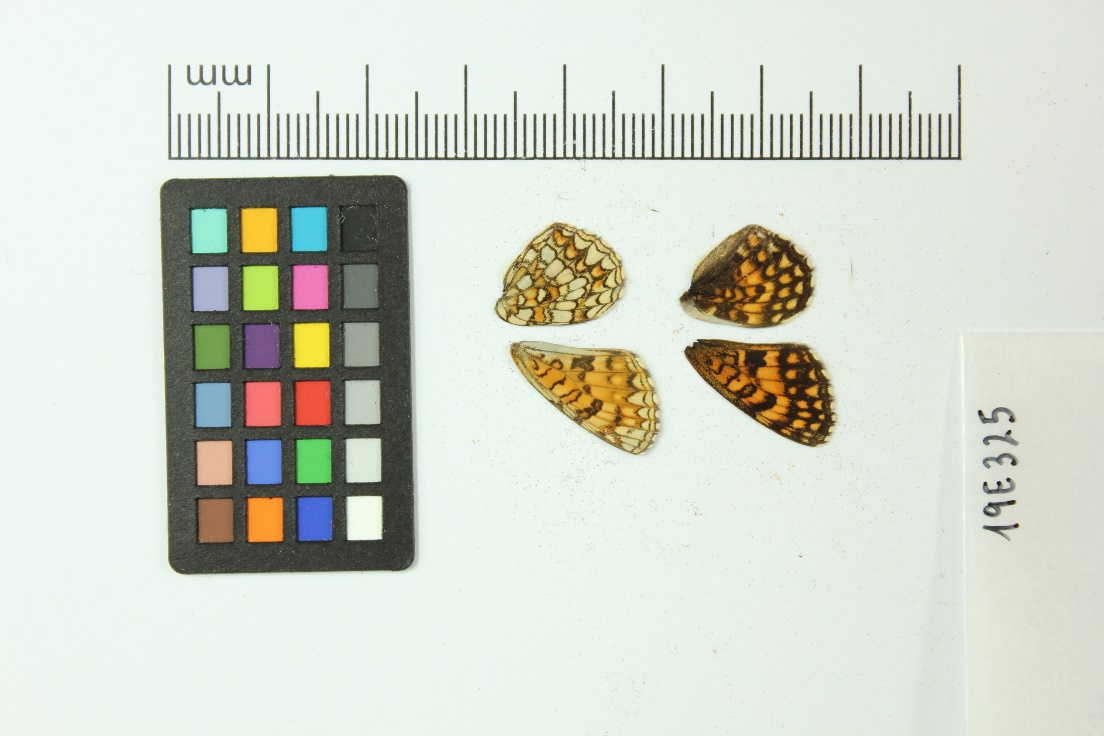


**M (paratype)**


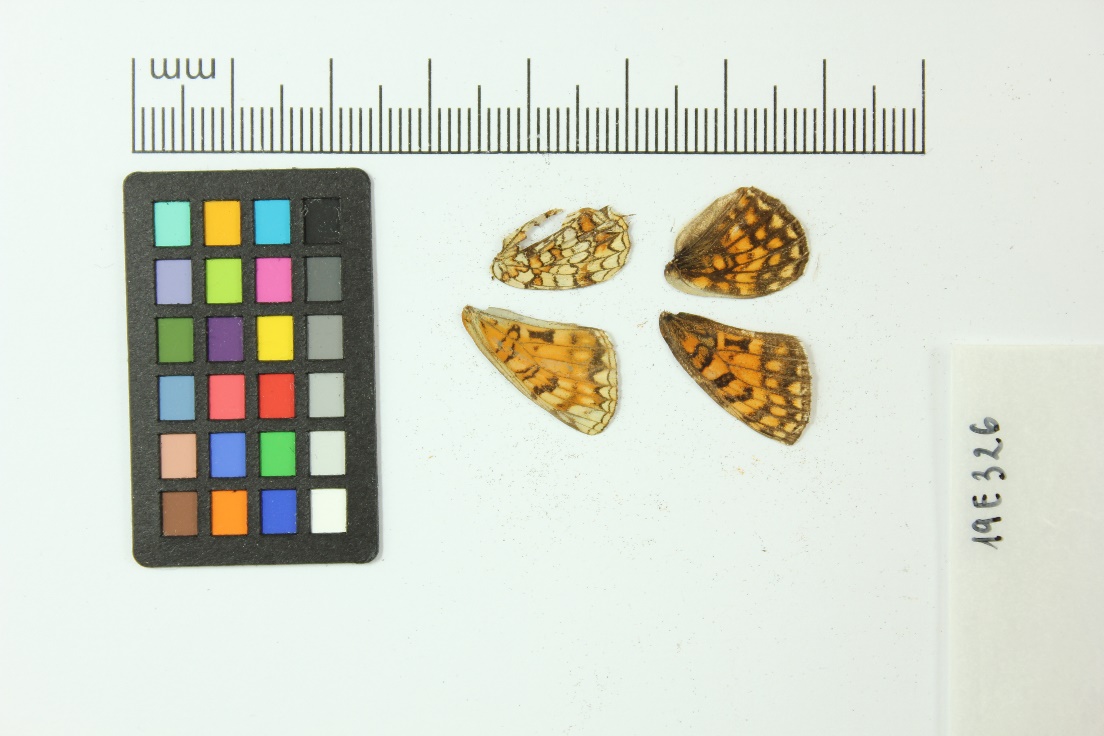


**N (paratype)**


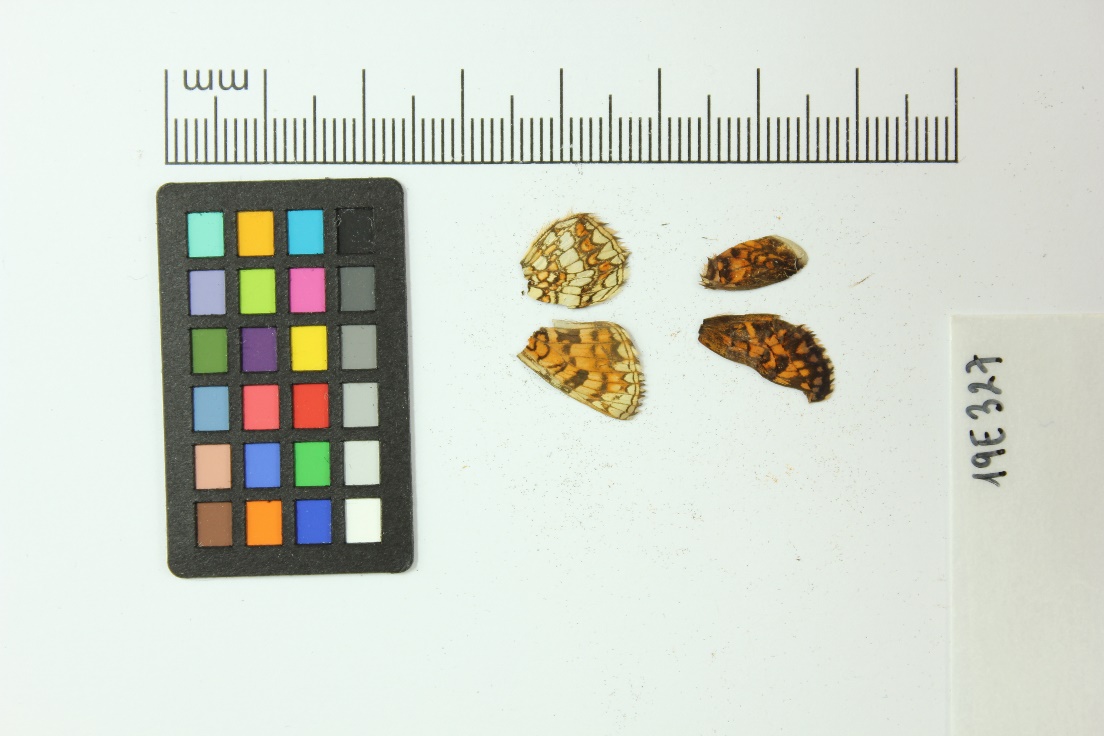


**O (paratype)**

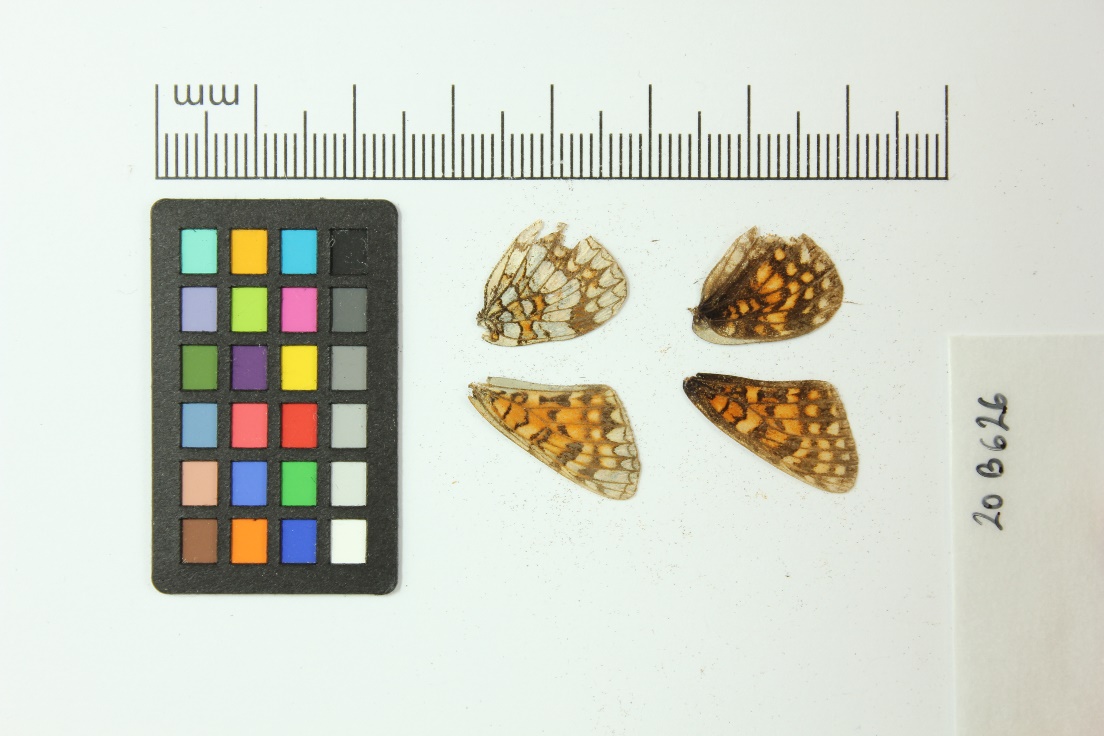


**P (paratype)**

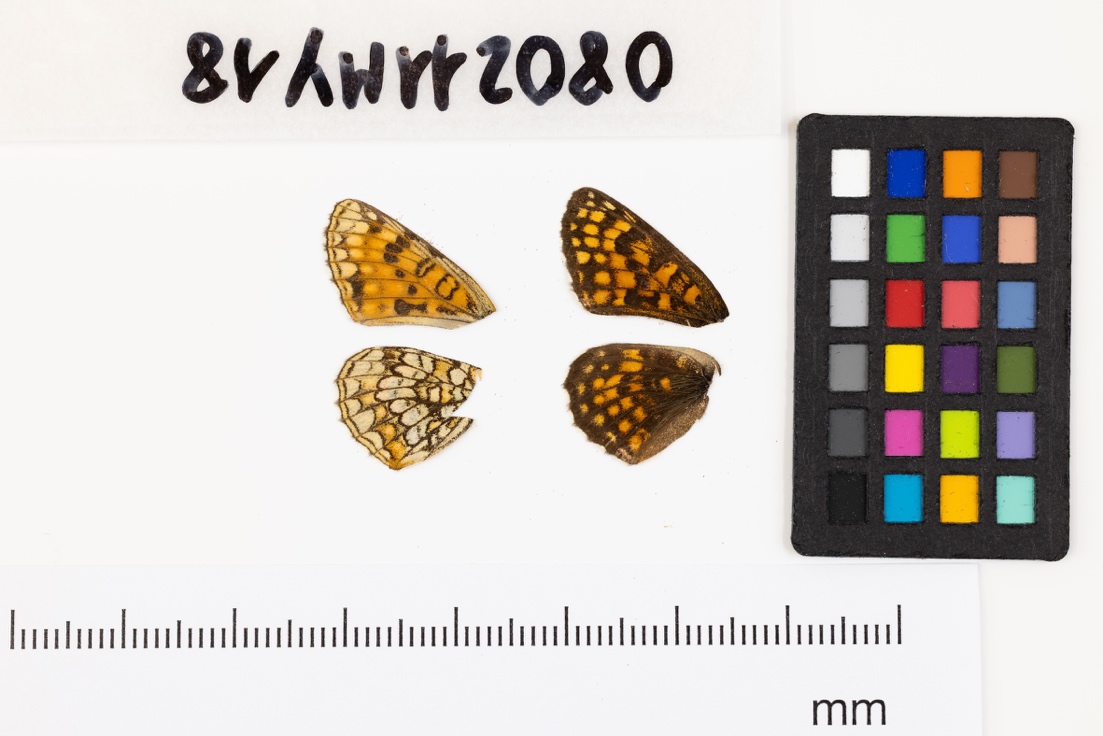


**A**


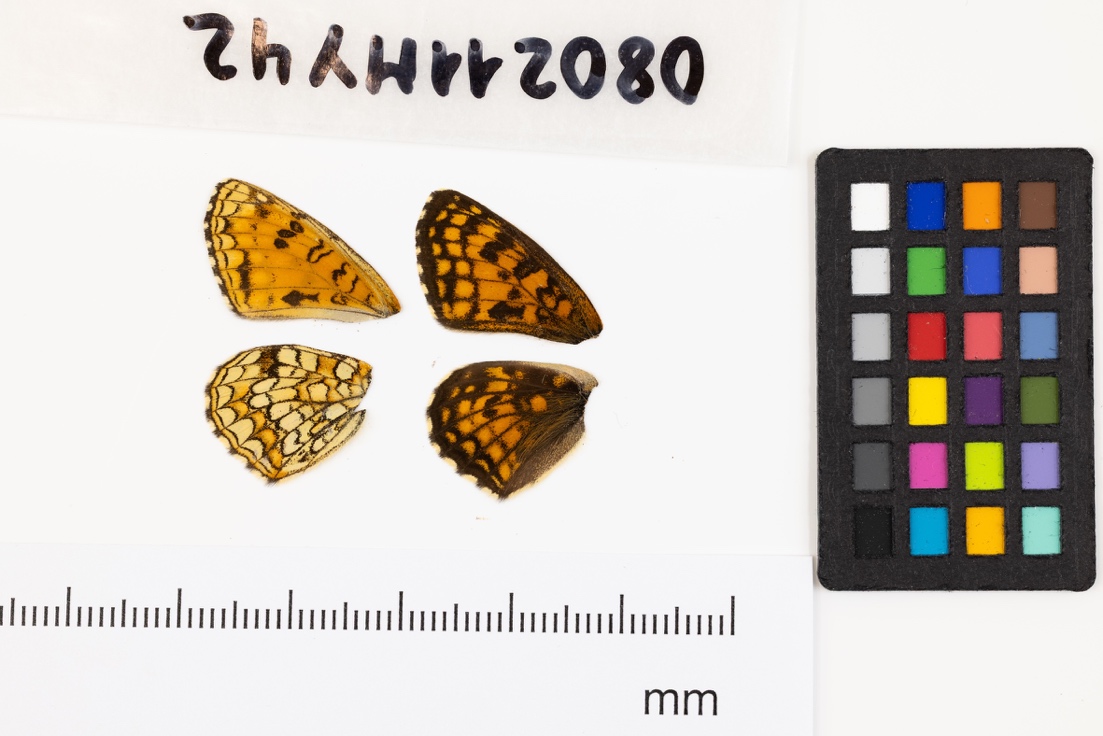


**B**


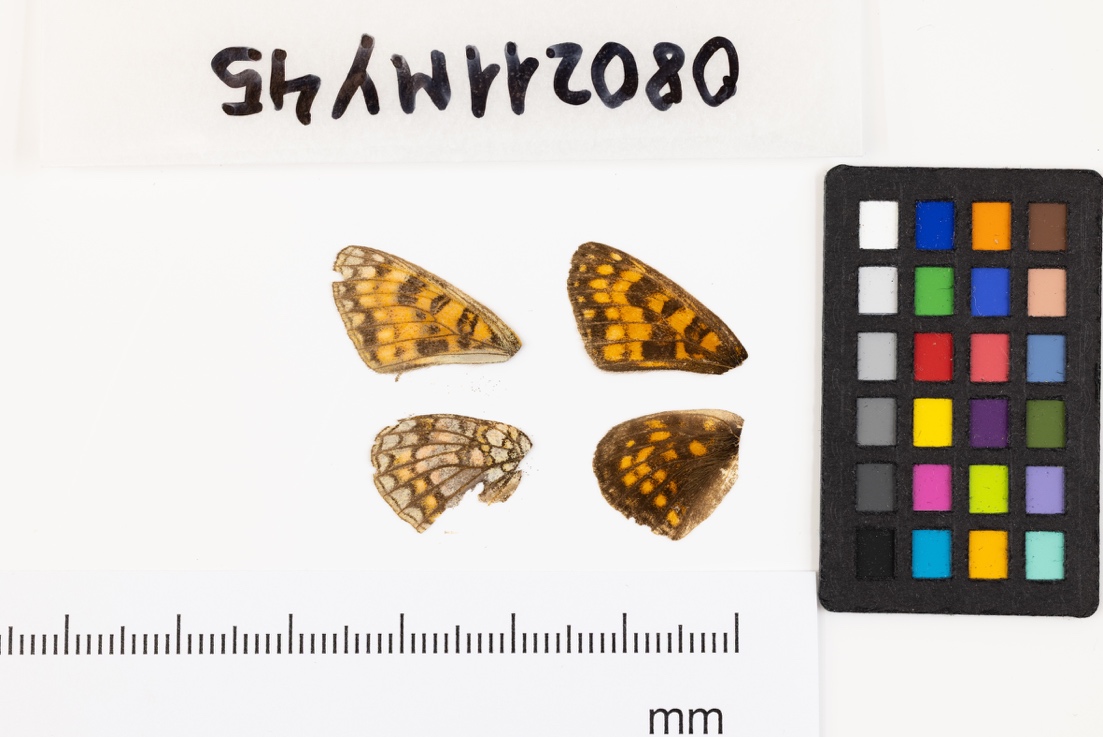


**C**

**Figure S9:** Wings of Melitaea diamina codinai ssp. reinst. specimens used in this study. Sample codes: (A) 080211MY18; (B) 080211MY42; (C) 080211MY45. Metadata in Table S1.


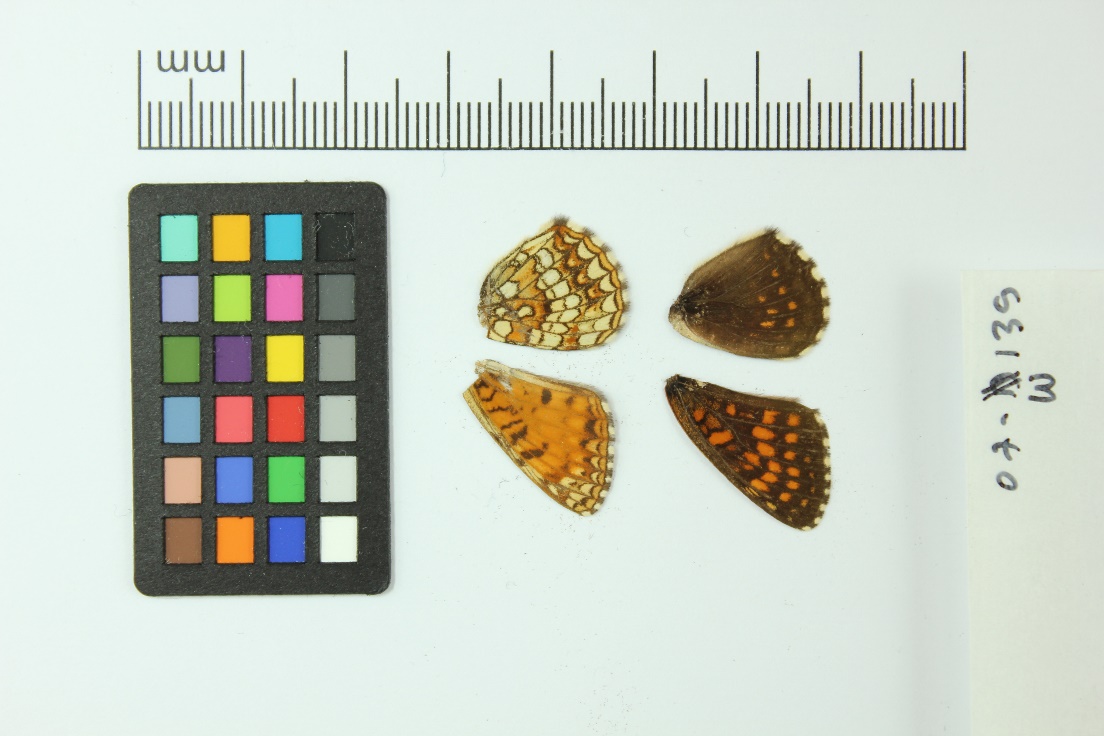


**A**


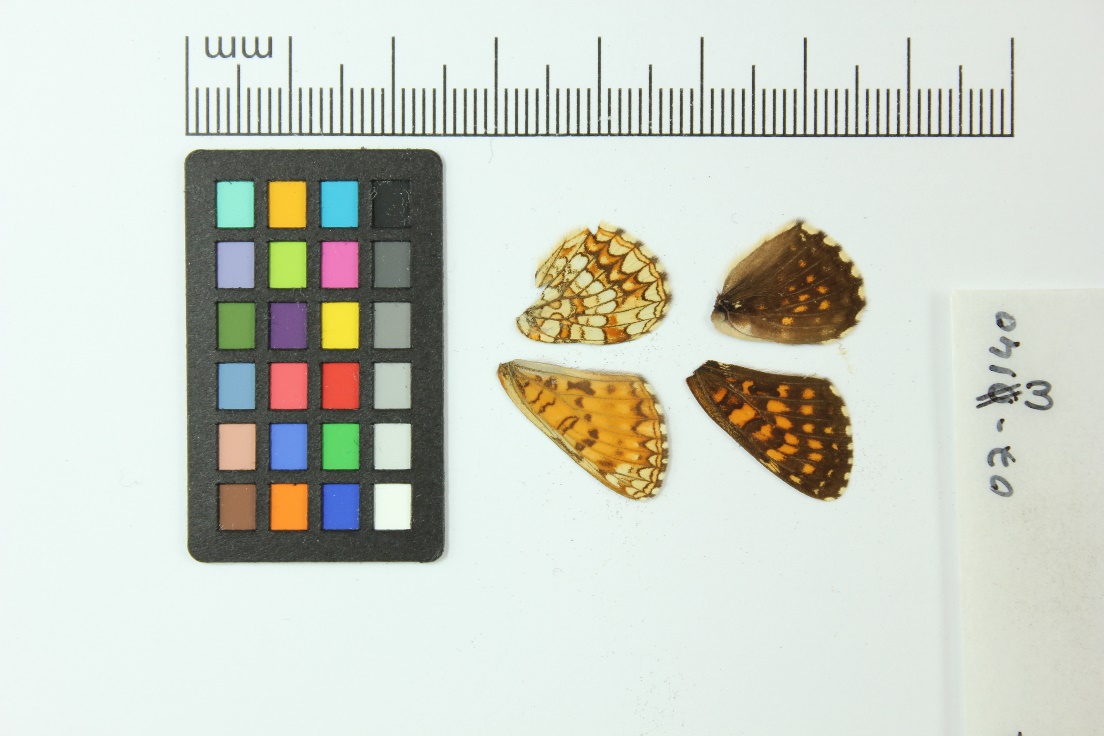


**B**


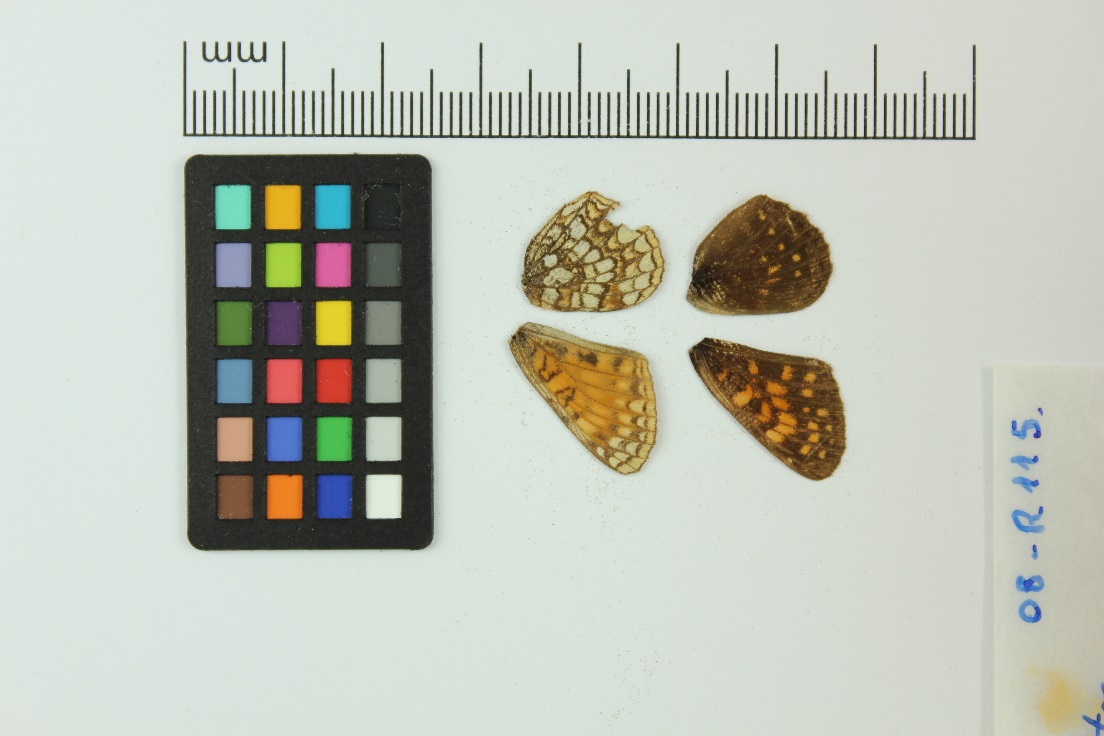


**C**


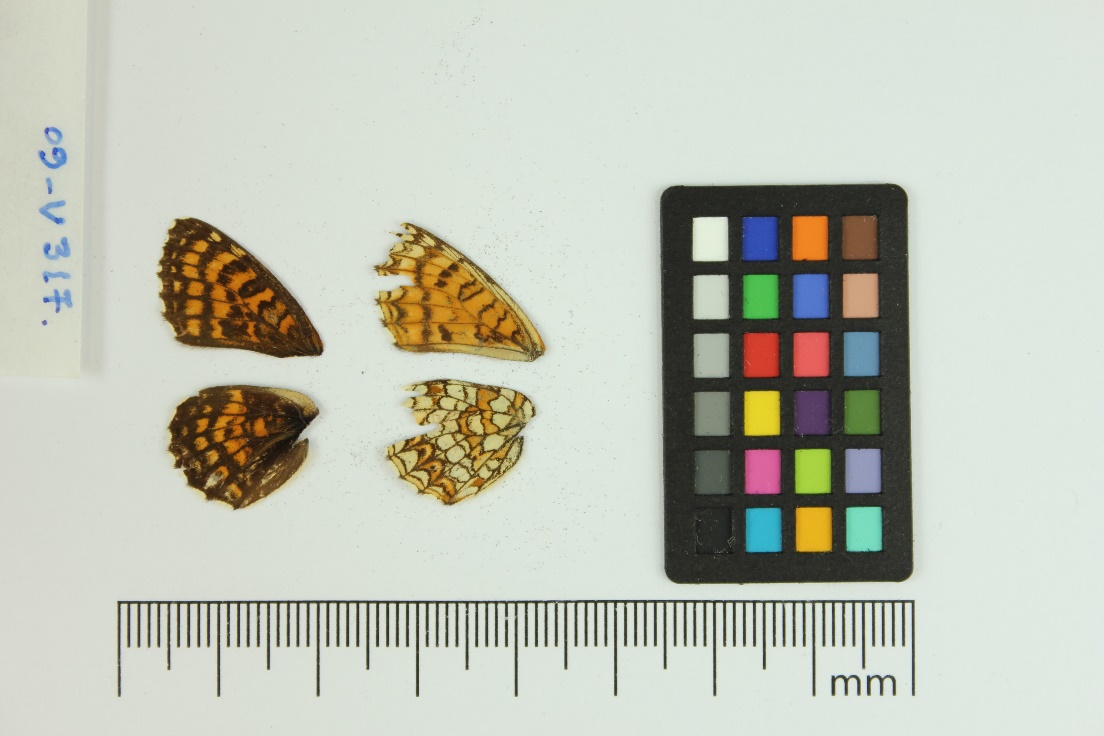


**D**

**Figure S10:** Wings of Melitaea diamina vernetensis ssp. reinst. specimens used in this study Sample codes: (A) RVcoll07W139; (B) RVcoll07W140; (C) RVcoll08R115; (D) RVcoll09V317; (E) RVcoll17B592; (F) RVcoll17E128. Metadata in Table S1, Supporting information.


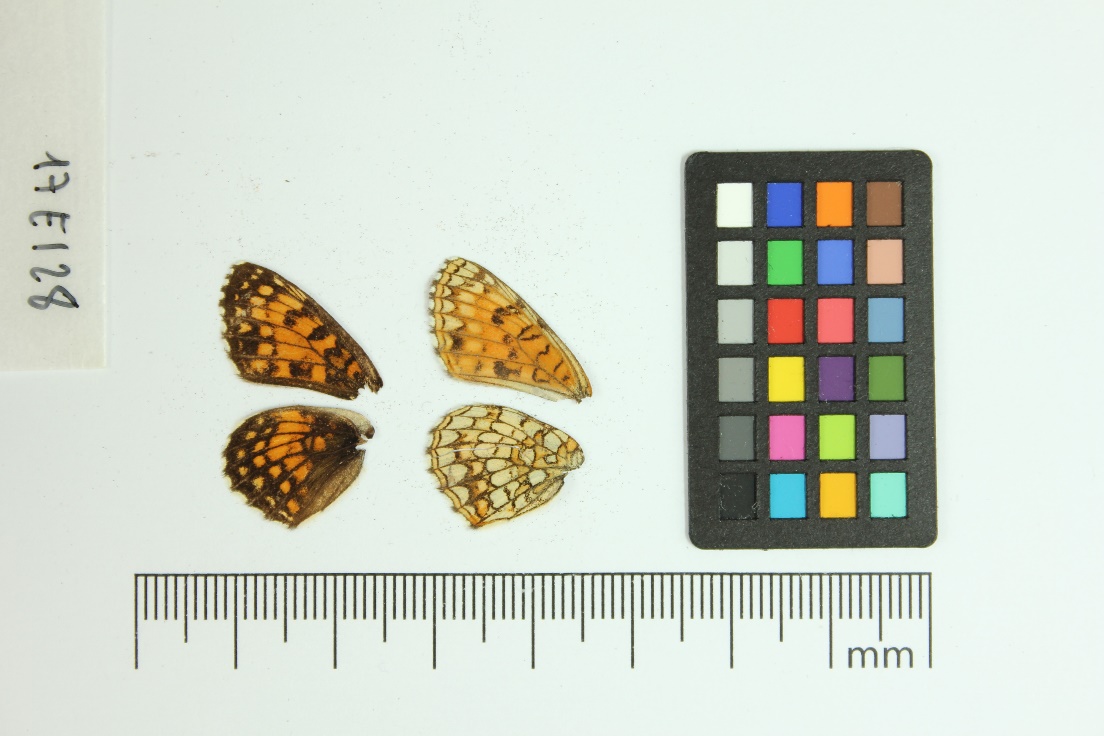


**F**


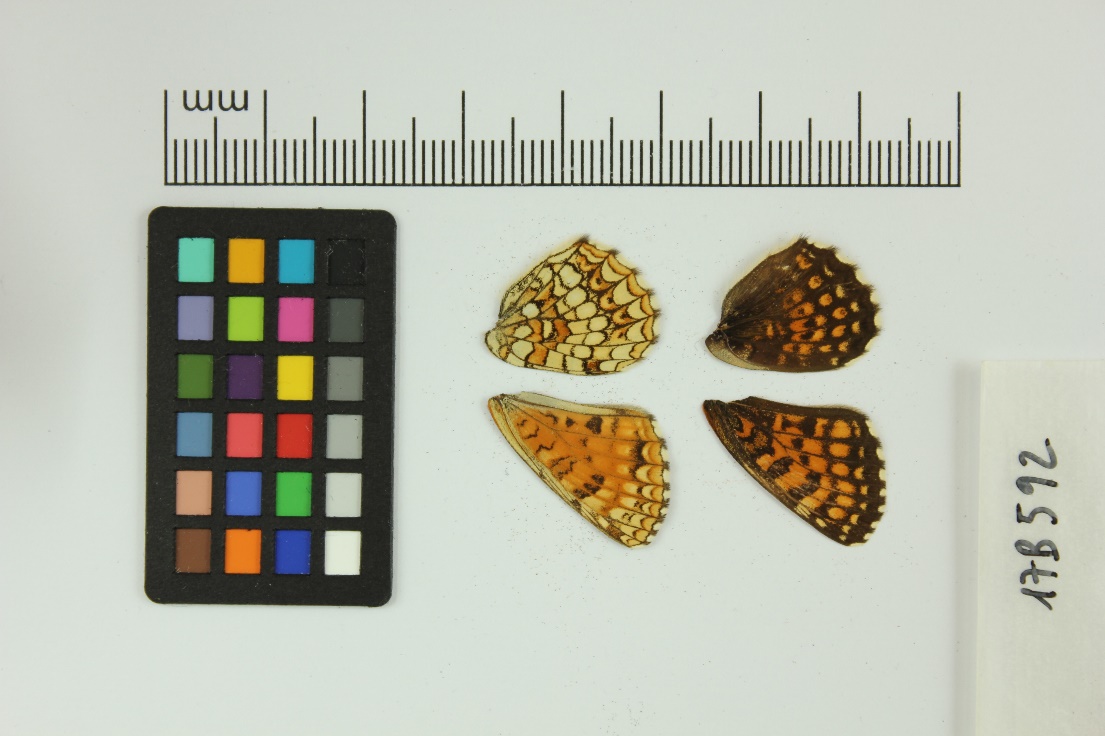


**E**
